# Identifying research priorities for improving information and support for patients undergoing breast cancer surgery: a UK patient-centred priority setting project

**DOI:** 10.1007/s10549-024-07413-8

**Published:** 2024-06-24

**Authors:** Emma Johnston, Katherine Cowan, Mairead MacKenzie, Sonia Patton, Lesley Turner, Patricia Fairbrother, Stuart A. McIntosh, Shelley Potter

**Affiliations:** 1 Patrick G Johnston Centre for Cancer Research, Queen’s University Belfast, 97 Lisburn Road, Belfast, BT9 7AE UK; 2 Katherine Cowan Consulting Limited, St Leonards-On-Sea, UK; 3 Independent Cancer Patients’ Voice, London, UK; 4 Bristol Surgery and Perioperative Care Complex Intervention Collaboration, Medical School, Translational Health Sciences, Bristol, BS10 5NB UK; 5 Bristol Breast Care Centre, North Bristol NHS Trust, Southmead Hospital, Southmead Road, Bristol, BS10 5NB UK

**Keywords:** Breast cancer surgery, Research priorities, Consensus, Information and support, Priority setting partnership, James Lind Alliance

## Abstract

**Purpose:**

To use robust consensus methods with individuals with lived breast cancer experience to agree the top 10 research priorities to improve information and support for patients undergoing breast cancer surgery in the UK.

**Methods:**

Research uncertainties related to information and support for breast cancer surgery submitted by patients and carers were analysed thematically to generate summary questions for inclusion in an online Delphi survey. Individuals with lived breast cancer experience completed two Delphi rounds including feedback in which they selected their top 10 research priorities from the list provided. The most highly ranked priorities from the survey were discussed at an in-person prioritisation workshop at which the final top 10 was agreed.

**Results:**

The 543 uncertainties submitted by 156 patients/carers were categorised into 63 summary questions for inclusion in the Delphi survey. Of the 237 individuals completing Round 1, 190 (80.2%) participated in Round 2. The top 25 survey questions were carried forward for discussion at the in-person prioritisation workshop at which 17 participants from across the UK agreed the final top 10 research priorities. Key themes included ensuring patients were fully informed about all treatment options and given balanced, tailored information to support informed decision-making and empower their recovery. Equity of access to treatments including contralateral mastectomy for symmetry was also considered a research priority.

**Conclusion:**

This process has identified the top 10 research priorities to improve information and support for patients undergoing breast cancer surgery. Work is now needed to develop studies to address these important questions.

**Supplementary Information:**

The online version contains supplementary material available at 10.1007/s10549-024-07413-8.

## Introduction

Breast cancer affects approximately 55,000 patients every years in the UK [1]. Although breast cancer treatment is multimodal, most patients have surgery. Breast cancer surgery, however, is increasingly complex. Patients are often presented with multiple surgical options including advanced oncoplastic procedures that may allow them to avoid mastectomy or, if mastectomy is required, a sometimes bewildering array of breast reconstruction options [2, 3]. Improvements in the use of neoadjuvant systemic anticancer treatment now offers patients the possibility of response adjusted surgery to both the breast and axilla [4], and the introduction of mainstream genetic testing had led to the identification of more gene carriers who face complex decisions about risk-reduction surgery [5].

Patients therefore need high-quality information and support to help them navigate their breast cancer diagnosis, make informed decisions about their treatment options, manage their postoperative recovery and any adjuvant treatments they may need and, if appropriate, decide how to manage their future breast cancer risk. As most patients will be long-term breast cancer survivors [6], understanding what information and support is required and how best to provide this is vital. A recent review, however, has highlighted that currently, women undergoing breast cancer surgery have unmet information and support needs [7] with further studies suggesting that younger women [8] and those considering more complex reconstructive surgery [9, 10] in particular, may lack the necessary information and support to make fully informed decisions regarding their care.

In 2022, over 200 patients and 100 healthcare professionals participated in a James Lind Alliance (JLA) Priority Setting Partnership (PSP) to identify the ‘top 10’ research priorities for breast cancer surgery in the UK [11]. These priorities included three questions related to the provision of information and support for patients with breast cancer and those at high genetic risk. Although inclusion in the ‘top 10’ highlighted the importance of the topic to both patients and professionals, the included priorities were extremely broad and their utility for informing future research was limited. Further work to identify specific research priorities related to information and support was therefore needed. Individuals with lived experience have an in-depth understanding of how information and support for breast cancer surgery is currently provided and how it could be improved. Exclusively involving patient partners in identifying research priorities to improve information and support would therefore ensure that the priorities generated were meaningful and reflected issues that were most important to patients.

The aim of this project was to extend the Breast Cancer Surgery JLA PSP [11] and use robust consensus methods to agree the top research priorities for improving information and support for breast cancer surgery with individuals with lived experience.

## Methods

This patient prioritisation process was conducted over an 8 month period between August 2023 and March 2024. As the project constituted patient and public involvement and engagement (PPIE) to identify and prioritize research questions, ethical approval was not required.

### Project advisory group (PAG) and project partners

Individuals with lived breast cancer experience, those at high genetic risk and families/carers were the main partners for this project. As this was an extension of the JLA PSP, a small project advisory group (PAG) was convened consisting of breast cancer surgeons involved with the main PSP who had extensive knowledge of the data set and appropriate methodological expertise, and patient advocates from UK patient advocacy group, Independent Cancer Patients’ Voice (ICPV). The project was overseen by an independent facilitator (KC) with specialised expertise in using consensus methods for research prioritization, ensuring a robust, transparent, and inclusive process.

### Scope

The scope of this patient prioritisation process was defined as information and/or support related to any aspect of breast cancer surgery which as per the JLA PSP encompassed ‘all areas of breast cancer care where breast surgeons were primarily involved in clinical management or where surgical input was central to multi-disciplinary treatment’ [11]. This included but was not limited to information and support:

- At diagnosis
- Around treatment and in particular surgical decision-making
- Around the time of surgery and recovery
- Longer-term information and support in the survivorship period
- Relating to risk-reduction surgery decision-making and the outcomes of risk-reducing surgery

Excluded were questions unrelated to breast cancer surgery, including those concerning aesthetic breast surgery in individuals without breast cancer, adjuvant breast cancer treatments including chemotherapy, radiotherapy, and endocrine therapy, as well as preclinical or basic science research related to breast disease. Decisions about whether questions were in or out of scope were made by the core study team (EJ, SP, SAMcI) and, if necessary, discussed with the PAG.

**Fig. 1:**
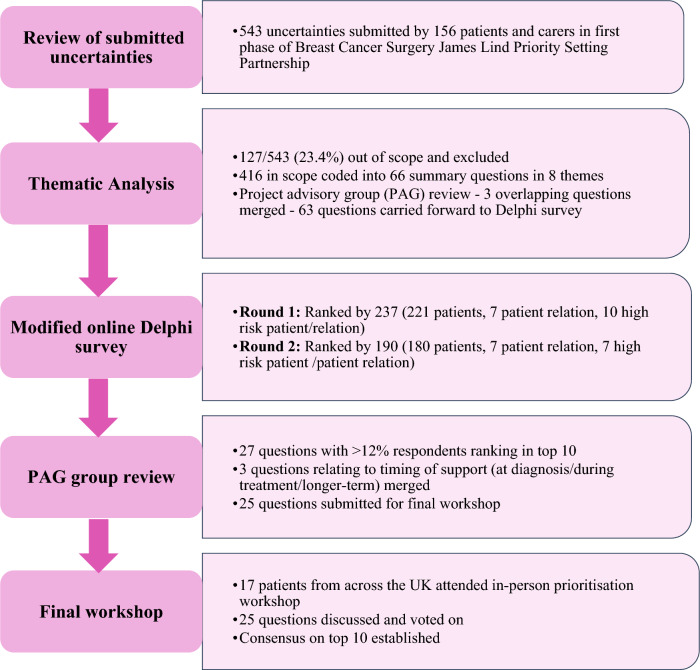
Flow diagram summarising the identifying research priorities for improving information and support for patients undergoing breast cancer surgery patient prioritisation project

The project consisted of three phases (Fig. 1):

1. Review of uncertainties submitted by patients/carers in the first phase of the Breast Cancer Surgery JLA PSP to identify themes related to information and support and develop of summary questions for inclusion in the Delphi survey
2. Two rounds of a modified online Delphi survey
3. An in-person prioritisation workshop with patient partners to agree the final top 10 research priorities for improving information and support for breast cancer surgery.

### Review of submitted uncertainties, identification of themes, and development of summary questions

Free-text uncertainties submitted by patients/carers participating in the first JLA PSP survey were extracted verbatim and the raw data reviewed in detail. Each submitted uncertainty was separated into its component parts so that they could be reviewed and analysed separately. Each question was coded as in or out of scope (OOS), with OOS questions excluded from further analysis.

Questions considered in scope were reviewed in more detail and analysed thematically [12]. An inductive/deductive approach was taken to the submitted uncertainties: for example, ‘what does a wire do?’ was coded as ‘information relating to the surgical procedure’. Summary questions were then drafted based on emerging themes and iteratively refined as analysis progressed. Emerging themes and proposed questions were reviewed by a second researcher with experience of qualitative methods to ensure that they were grounded in the data and reflected the uncertainties raised by patient participants. The questions were reworded and revised based on feedback from patient advocate members of the PAG to ensure that they were understandable by a broad audience prior to inclusion in the Delphi survey. Evidence checking was undertaken as part of the JLA PSP. This focussed on high-quality data sources to identify the most relevant and up-to date evidence. The selected data sources were (i) UK guidelines; (ii) the Cochrane database of systematic reviews; (iii) Early Breast Cancer Clinical Trialists’ Collaborative Group (EBCTCG); and (iv) Targeted PUBMED searches [11]. Only guidelines or reviews published in the last 5 years were included to ensure they reflected the most up-to-date evidence in each area. Questions were considered answered if recent (within the last 5 years) systematic reviews identified moderate or high-quality evidence to address the topic. As no relevant guidelines updates or additional high-quality evidence (specifically, large-scale multicenter randomized trials) were identified as having been published or presented in the interim, a further detailed search was not considered necessary.

### Modified online Delphi survey

Individuals with lived breast cancer experience and those at high genetic risk together with their families and carers were invited to participate in two rounds of an online Delphi survey to identify the top research priorities for improving information and support for women undergoing breast cancer surgery.

The survey was developed using SurveyMonkey software and included an introduction to the project, specific instructions regarding the importance of completing both rounds of the survey to establish consensus and a list of the questions generated from the survey data. Questions were presented in a random order and participants asked to select their top 10 from the list provided. Simple demographic information including age, geographical location and ethnicity was also collected. Participants were asked to provide a valid e-mail address for distribution of the round 2 survey and to express an interest in participating in the final in-person prioritisation workshop. The round 1 survey was open between 11/01/23 and 12/08/23 and was circulated widely via social media, patient advocacy groups and breast cancer charities to optimise engagement and participation.

All top 10 questions selected by respondents were given one point and the total score for each item calculated. The number and percentage of respondents including each round 1 question in their top 10 was summarized and fed back to participants in the round 2 survey.

All respondents who provided a valid e-mail address in Round 1 were invited to participate in Round 2. This comprised the same set of questions presented with a random order together with feedback from round 1. Participants were asked to review the questions again and choose their top 10 taking into account the feedback received. The round 2 survey was open between 12/29/2023 and 02/05/2024 and a reminder e-mail was sent to non-respondents 2–3 weeks after the initial invitation to optimise response rates.

Round 2 responses were scored in the same way as in round 1 with one point given to each item included in the top 10 and the total score used to determine the overall rank. Final rankings were reviewed by the core team (KC, SP and SAMcI) to determine which questions should be carried forward to the prioritisation workshop.

### Final prioritisation workshop

Survey participants expressing an interest in taking part in the final in-person workshop were purposively invited to participate based on age, geographical location, and lived breast cancer experience to ensure their views were as representative of the UK breast cancer community as possible. Confirmed participants were asked to declare any interests or conflicts, and to submit a biography.

Prior to the workshop, all participants were sent information packs containing preparatory information and all attendees’ biographies, and asked to identify their ‘top three’ and ‘bottom three’ research priorities from the list provided.

The in-person workshop consisted of two rounds of discussion and voting with expert facilitation. During the workshop, taking an approach similar to that of the JLA PSP workshop, participants were divided into small groups of 5–6 individuals with differing experiences of breast cancer surgery and a facilitator. Following introductions, the facilitator asked each individual in turn to list their top and bottom three priorities and discuss the rationale for their choices. Once all individuals had shared their views, wider group discussions were encouraged with the facilitator ensuring each individual had the chance to participate, and maximising the opportunity for knowledge exchange and shared learning. Participants were then allocated to a new small group and again asked to share their top and bottom three research priorities, reflecting on whether or not these had changed following previous discussions. Workshop participants were then asked to anonymously select and rank their top 10 priorities for information and support research using online voting software and the final results presented to the whole group.

### Feedback following presentation of top 10 research priorities

Following the in-person workshop, participants were sent an online survey asking for reflections on the process and their views on the top 10.

## Results

The project has been reported according to REPRISE guidelines [13].

### Review of submitted uncertainties, identification of themes, and development of summary questions

A total 543 uncertainties were submitted by 156 individuals with lived breast cancer experience and/or their carers in the initial phase of the JLA Breast Cancer Surgery PSP. Of these, 127 (23.4%) were considered OOS and excluded. The remaining 416 (76.6%) were reviewed and categorised into 66 summary questions in eight key themes. These included (i) information and provision of support (n = 14); (ii) neoadjuvant therapies and treatment sequencing (n = 4); (iii) breast cancer surgery (n = 8); (iv) breast reconstruction (n = 11); (v) contralateral mastectomy and flat symmetry (n = 7); (vi) surgery and post-operative care (n = 13); (vii) long-term follow-up and support (n = 5); and (viii) high risk patients and special groups (n = 4). Details of the submitted uncertainties and summary questions within each theme can be found in Online Resource 1. Following review by the PAG, three questions were considered to overlap and were merged. A total of 63 questions were therefore included in the Delphi survey.

### Modified online Delphi survey

A total of 237 individuals completed the round 1 survey and of these 190 (80.2%) participated in round 2. Most respondents had lived breast cancer experience, were female, white, and lived in England (Table 1).

**Table 1:** Demographics of Delphi participants

	Round 1 (n = 237, %)	Round 2 (n = 190, %)
Lived breast cancer experience		
Person being treated/previously treated for breast cancer	220 (92.8)	180 (94.7)
Partner/carer of a person with breast cancer/at high genetic risk	9 (3.8)	4 (2.1)
Person at high genetic risk of breast cancer	8 (3.4)	6 (3.2)
Age of respondents		
Under 40	22 (9.3)	8 (4.2)
41–50	51 (21.5)	38 (20.0)
51–60	87 (36.7)	60 (31.6)
61–70	57 (24.1)	52 (27.4)
Over 70	15 (6.3)	10 (5.3)
Not reported	6 (2.5)	22 (11.6)
Age of respondents at diagnosis		
Under 40	33 (13.9)	16 (8.4)
41–50	82 (34.6)	67 (35.3)
51–60	77 (32.5)	52 (27.4)
61–70	27 (11.4)	24 (12.6)
Over 70	4 (1.7)	3 (1.6)
Prefer not to say/not reported	14 (5.9)	28 (14.7)
Geographical location		
England	180 (75.9)	132 (69.5)
Wales	12 (5.1)	10 (5.3)
Scotland	24 (10.1)	18 (9.5)
Northern Ireland	15 (6.3)	8 (4.2)
Prefer not to say/not reported	6 (2.5)	22 (11.6)
Ethnicity		
White	229 (96.6)	166 (87.4)
Other	4 (1.7)	2 (1.1)
Prefer not to say/not reported	5 (2.1)	22 (11.6)
Gender		
Female	231 (97.5)	169 (88.9)
Male	3 (1.3)	1 (0.5)
Prefer not to say/not reported	4 (1.7)	20 (10.5)

The number of participants including each of the 63 questions in their top 10 research priorities in rounds 1 and 2 is detailed in Online Resource 2. Following Round 2, the proportions of participants including each question in their top 10 research priorities were reviewed to determine which questions should be carried forward for discussion at the final prioritisation meeting. A clear cut off was identified with 27 questions included in the top 10 by more than 12% of respondents. This list was reviewed and three questions all related to the timing of support (at diagnosis, during treatment and long-term) were identified. Following discussion with the study team, these questions were merged into a single item focussed on psychological and emotional support throughout the breast cancer journey (Online Resource 2). A total of 25 questions were therefore carried forward for discussion at the final prioritisation workshop (Online Resource 3).

### Final prioritisation workshop

The in-person workshop was attended by 17 participants with diverse experiences of breast cancer treatment. Participants included younger and older women treated for breast cancer and those at high genetic risk with broad geographical representation from across the UK. Following two rounds of discussion, participants voted anonymously on their top 10 research priorities. The final top 10 are shown in Table 2. These were presented to the participants who agreed that these priorities reflected their discussions.

**Table 2:** Top 10 research priorities for improving information and support for women undergoing breast cancer surgery

Rank	Priority
1	How can we ensure that all patients with breast cancer are offered and given fair and balanced information about all appropriate types of breast cancer surgery? How can we best support patients to make decisions about what option is best for them?
2	How can we support healthcare professionals to routinely offer balancing mastectomy for symmetry as an alternative to breast reconstruction in patients who do not carry a breast cancer gene who have had a single mastectomy for breast cancer?
3	How can we support patients to be fully involved in decisions about their breast cancer treatment? How can this support be tailored according to the patient’s wish to be involved?
4	How can we best tailor information about breast cancer treatments for individual patients so that it is personalized for them and their circumstances?
5	How can we best prepare patients for breast cancer surgery including aftercare (e.g. bras and dressings) and what to expect after the operation? What information do patients want and how should this be provided?
6	What are the short- and long-term cancer-related outcomes after breast conserving surgery and radiotherapy vs mastectomy? Which operation has the lowest risk of the cancer returning?
7	In patients having mastectomy for breast cancer who do not carry a breast cancer gene, should a balancing mastectomy for symmetry be offered at the same time or at a later date? What factors may influence this decision?
8	How many women get lymphoedema after breast cancer surgery? Can it be prevented? What is the best way to detect and treat lymphoedema if it develops?
9	Why is there variation in the types of breast cancer treatment (including surgery) offered in different areas of the UK and how can this be addressed?
10	What are the short and long-term outcomes of mastectomy surgery and how does mastectomy affect patients’ quality of life?

### Post-workshop feedback

The post-workshop online survey was completed by 15/17 (88.2%) participants. Having reflected on the top 10, the majority agreed that these were representative of workshop discussions. Several highlighted a degree of overlap within the top 10 priorities and how the terminology could be improved. Others emphasised that all the questions discussed were important to patients and should be considered future research priorities.

## Discussion

This project has used robust consensus methodology to agree the top 10 research priorities for improving information and support for patients undergoing breast cancer surgery with individuals with lived breast cancer experience. It has highlighted the need to ensure that patients are given balanced and personalized information about all treatment options and appropriate support to allow them to be meaningfully involved in decisions about their care. High-quality information to prepare women for surgery and support them to effectively manage complications including lymphoedema were identified as important areas for future research. Ensuring equity in access to all treatment options, including supporting clinicians to offer contralateral symmetrizing mastectomy as an alternative to breast reconstruction was also a key research priority.

These priorities complement the existing top 10 [11] by providing clear insights into the research needed to improve information and support for patients undergoing breast cancer surgery. The information and support priorities are particularly meaningful as they have been agreed in partnership with individuals from across the UK with a diverse range of lived experiences of breast cancer treatment. Despite this diversity, however, issues related to the lack of equity of choice, access to treatment options, appropriate information, and support for women to actively participate in decisions about their care resonated through group discussions highlighting that such issues are widespread and urgently need to be addressed. Although some concerns, particularly those relating to equity of access have already been raised by charities [14] and parliamentary groups [15], this is to our knowledge the first time that patients with experience of breast cancer have independently identified these areas as key research priorities. As such, these information and support research priorities provide a powerful mandate for researchers to work in partnership with patients to design studies that will directly and meaningfully improve the experiences and outcomes of women undergoing breast cancer surgery in the future.

Although robust consensus methods were used to agree the top research priorities, this work has limitations that require consideration. Firstly, although every effort was made to recruit a diverse range of participants through social media, charities, patient groups, and advocacy networks, the majority of respondents were white. The lack of inclusion of ethnically and culturally diverse women is potentially problematic as the challenges experienced by these groups may differ, and therefore, the top 10 agreed here may not reflect their priorities or concerns. Targeted recruitment of patient advocates from more diverse communities will therefore be essential when developing future research to ensure that studies are designed to reflect the needs of all patients with breast cancer. Women at high genetic risk and men were also underrepresented in the process but these groups make up a much smaller proportion of breast cancer patients. However, as the questions prioritized focussed on fundamental issues including equity, optimizing choice, informed consent, and patient-centred care, the research generated is likely to benefit all patient groups.

One of the main concerns raised by workshop participants was that several of the questions overlapped, potentially resulting in questions covering a broader range of issues being prioritized over those that were more specific. This was directly addressed in the workshops by providing clear instructions that participants should prioritize the questions that mattered to them and not assume that they would be covered by a broader question. Information, support, and decision-making, however, are intrinsically linked so clear separation of issues is conceptually challenging, and despite these issues, the workshop participants were satisfied that the top 10 agreed at the end of the workshop reflected the views of the group.

High-quality research is now needed to address these research priorities and improve the information and support for patients facing breast cancer surgery in the future. While multiple decision aids have been developed to support specific aspects of breast surgical decision-making [16–18], these have not been widely implemented into practice. Furthermore, they are limited in scope and are unlikely to offer the personalized information and support for all the aspects of the breast cancer pathway highlighted here. There is therefore a need to co-develop and evaluate new resources in collaboration with individuals with lived experience so that these meet the needs of patients facing decisions about breast cancer surgery in the future. These will need to be flexible and digital platforms may offer the ideal opportunities to develop such resources. Interventions are also needed to address issues such as equity and access. These interventions are likely to be complex as they will involve multiple key stakeholders including [19] commissioners and health care providers as well as patients and clinicians. Innovative study designs and novel methodological approaches such as realist evaluation [20] and process mapping [21] are likely to be essential to develop and evaluate these new processes and pathways of care. Stakeholder engagement will be essential for future implementation but patients must be central to the design and development of all future studies if they are to generate truly meaningful interventions. The top 10 priorities to improve information and support for patients undergoing breast cancer surgery have set the research agenda. Patients, clinicians, methodologists, and other key stakeholders will now need to work together to design and deliver high-quality studies to allow these important questions to be addressed.

## Supplementary Information

Below is the link to the electronic supplementary material.

Supplementary file1 (DOCX 118 KB)

## Data Availability

All data generated during this project are presented in the manuscript and online resources.
