# Supplementary material for: Identifying research priorities for improving information and support for patients undergoing breast cancer surgery: a UK patient-centred priority setting project
Source: Breast Cancer Res Treat. 2024 Jun 24;208(1):215–22. doi: 10.1007/s10549-024-07413-8 (PMC11452454; doi:10.1007/s10549-024-07413-8)
Supplement: Supplementary file 1 — Supplementary file1 (DOCX 118 KB) [file 10549_2024_7413_MOESM1_ESM.docx]

**Online resource 1: Themes, submitted uncertainties and summary questions for information and support for breast cancer surgery**

| **THEME 1: INFORMATION AND PROVISION OF SUPPORT** | | |
| --- | --- | --- |
| **Submitted uncertainty/raw data** | | **Summary question** |
| **B2.7** | Is information about the pros and cons of each option easily accessed? | What information do patients want to help them make decisions about their breast cancer treatment options and how should it be provided? |
| **A112.1** | What is the best way for a patient to find out more information; have questions answered. |  |
| **B112.6** | Best treatment for recovery time |  |
| **A120.2** | How much literature is available about treatment choices? |  |
| **B90.10** | Information To help patients make a more informed decision about which surgical procedure to choose, to manage their expectations, and to help make them feel more supported: What is the best way to provide patients with information on the different types of surgical options, what's involved in each, and before and after images of each? |  |
| **A74.2** | I think that once pathology has been confirmed and your 'type' of cancer is known, this info should be given to you in a written format and I think that more detail would be useful for the patient. It shouldn't take a patient asking many months or even a year down the line for more info on their diagnosis. Hormone status, Her2 status, ductal, lobular etc...all this info should be available and even the scores. For example, if a patients has an 8/8 estrogen score this may increase understanding of risk and later drug adherence to anti- estrogen meds. I think that some degree of uniformity of information delivery would benefit patients. I feel that sometimes patients are at the mercy of a consultant or doctor's personality, perspective on risk, experience and manner in which they can relay info. Some are very statistic driven, some can drip information while they assess how much they think you should know, some can make a surgery decision with very little consultation of the patient's wishes or fail to explain why a particular surgery decision has been made and what this means for your chance of recurrence or risk of MBC. I think that this can be very problematic and can lead to major emotional strain for the patient. |  |
| **A21.1** | Information on the choice of tests and treatments is critical. I missed being able to have an Oncotype DX test as my consultant never mentioned it. |  |
| **B127.3** | Would it be possible to have a model (like NHS Predict) into which you could put your breast cancer details and see the percentages of survival / risk of recurrence/ need for further surgery for different options of surgery, timing in relation to other treatments. e.g. If you are offered chemotherapy first to shrink the lump what are the chances that i would lead to a lumpectomy rather than a mastectomy? |  |
| **A148.1** | How to provide full accurate information Where is the best place for me, with my individual wants and needs, to be treated | How can we best tailor information about breast cancer treatments for individual patients so that it is personalised for them and their circumstances? |
| **A168.1** | How much information do people want about their possible treatments - all up front or on a ‘need to know’ basis? |  |
| **A78.2** | *it is information that is required but as and when it is relevant for each step and as I can take it in. Good, clear information and contact to ask further questions. Not bombarded with leaflets * what tests and scans are needed and WHY * surgery type and why that recommendation |  |
| **A125.1** | I understand that different people want different levels of information, but work could usefully be done on establishing that at the outset for each patient, then identifying sources of information depending on how much the individual wants to knw. I spent a lot of time trying to find stuff out for myself (particularly in relation to staging, grading, treatment options and prognosis) because cinicians didn't seem interested in talking about these things. |  |
| **B160.2** | Patients views should be foremost in everyone’s minds. Being asked to make life changing decisions at a distressing and frightening time needs support. | What support do patients want to allow them to be fully involved in decisions about their breast cancer treatment? How can this support be tailored according to the patient’s wish to be involved? |
| **B126.2** | Will surgeons/breast care specialists please take more time to explain the rationale behind decisions which are made at team meetings. Why can they not be more open about the options? |  |
| **A99.1** | Is it better to offer diagnosis and treatment planning in the same appointment, or separately? - do people find it hard to make decisions when they have just been given a difficult diagnosis? or can they cope better with the diagnosis if they've had a chance to make clear treatment plans? or is it different for different people? |  |
| **B35.3** | Lack of patient choice and minimisation of patient voice regarding mastectomy surgery options. |  |
| **B57.4** | What difference will a delay to seek second opinion or to consider all options make? |  |
| **B111.2** | To have time with the surgeon to understand fully all implications of surgery and what is meant by all options truthfully. For lobular that means accepting and explaining its uniqueness and why there may be need for further surgery. Explain reconstruction at a deeper level over a few appointments give people a chance where possible to digest info |  |
| **B141.2** | I asked my surgeon for a mastectomy and was told that it wasn't necessary and I had a wide local excision. I know that from pathology of the tissue removed there wasn't a clear margin. I understand a surgeon will try to preserve as much of the breast tissue as possible but should the patient have more of a say in how much tissue is removed (I would have been happier to have more removed). |  |
| **B15.3** | How can we prepare patients properly for DIEP reconstruction? I was not aware that my stomach area would be permanently numb. When I thought I had a small tumour on the right I opted for a mammoplasty. I underwent two breast preserving surgeries previous to my completion mastectomy with immediate DIEP. I would have opted for the double mastectomy from the start if I’d found out I had a tumour in each breast following my initial appointment. I attempted to say this when my bilateral diagnosis (IDC over 2cm in left and over 5cm in right) was confirmed by MRI a month after I presented with symptoms. However, my voice got lost as the surgeon and team were focussed on the breast preserving surgery I initially thought I wanted. I regret having to go through the mammoplasty then a re-excision neither of which got clear margins before the decision was made that I should have a mastectomy. |  |
| **A168.2** | How much say do people want in decisions about their treatment plan? |  |
| **B124.2** | More explanation & choices of the treatment plan, a kinder view. |  |
| **B33.2** | There should be enough time to discuss all options and maybe the timescales should be flexible if a patient needs to get further advise or delay this so that they can make the RIGHT decision for them instead of feeling pressurized by time and pushed along the conveyor belt... |  |
| **A143.1** | An understanding of the timelines, how long can we take with our decision making so that we are able to consider all options. |  |
| **A175.1** | Allow time for decision making. Do not bombard patients with everything at once. Listen to the patient. |  |
| **A179.1** | There is a lot of information to process when someone receives a diagnosis. How can we ensure patients and carers fully understand the pathway and timeline of the treatment we've agreed on? |  |
| **A80.2** | What follow up is there post appointment at the point of diagnosis. The patient normally takes a lot of information in but does not necessarily understand it all - they then return home and discuss with others who ask them questions which they do not have answers for. A few days after they feel clearer and may have further questions. |  |
| **A55.1** | What information do patients want at diagnosis and how should this be provided e.g leaflets or talking and opportunity to ask questions later |  |
| **A110.5** | What is the best information to provide at the time of diagnosis? Consistency around the format of what & how information is given to patients is essential. I have never received any information in written format along my cancer journey. A written individual treatment plan/log would have been extremely helpful. |  |
| **A146.1** | Try and get info across on diagnosis - knowing I could have a reconstruction immediately made a huge difference. Waking up with a cleavage was hugely important to My feeling normal. Not everyone can take in info so surgeon needs to try and understand what is important or have knowledgable nurse. Unfortunately I know when there is bad news when the nurse is her - but like her and she is helpful! |  |
| **A49.1** | How can you best prepare a person for the news that they have cancer? Are you giving clear and precise information to the person? Do you think that they are able to understand what you are telling them? When a person goes for a mammogram how can you prepare them for the possibility that they might need further tests and explain what those involve. Do you think the timeline between testing, diagnosis and communication of a diagnosis of cancer is reasonable? How do think the person experiences the waiting time between testing and diagnosis? You are making a treatment plan, do you think your patient should be involved in the planning? |  |
| **B2.5** | This decision should not be rushed into. What questions are patients asked to determine the best option {choice & timing of breast surgery} for them? |  |
| **A37.1** | Would you like some time to consider your options? |  |
| **A49.4** | You are making a treatment plan, do you think your patient should be involved in the planning? |  |
| **B176.2** | Patient choice, is it working in practice? |  |
| **A44.1** | It is overwhelming on being diagnosed especially when you don't really know anything about cancer. I had no idea I would have so many appointments in the first week. My oncologist was not upbeat. Mr X my consultant was fantastic, he looked after my mental health as well as physical. A breast care nurse wrote out an estimated time line of treatments for me which really helped. You really need some one with you as you never take in all that is said. You need to feel you are important and not a number and you need hope. | What practical and emotional support do patients with breast cancer want at diagnosis and during treatment? |
| **C165.4** | Having someone to talk to and go though any concerns especially directly after surgery while awaiting results of what comes next |  |
| **A151.1** | I had bilateral breat cancer Explaining what is about to you and why. Went through a number of procedures before even the surgery but not always sure what exactly was happening to me and why. I simply surrendered to their better judgement |  |
| **A45.1** | Was there support if you decided against surgery? |  |
| **C73.7** | I particularly wanted to know as soon as possible what follow up treatment I would need and whether I would need chemotherapy. |  |
| **A140.1** | choice, order and timeliness of tests and treatments |  |
| **A53.1** | Why is it ok to wait for over a month for surgery? |  |
| **A30.4** | Who, when, how to best support the patient with a breast cancer diagnosis - rehabilitation, post-habilitation. |  |
| **A78.1** | *At the point of diagnosis is the time when nothing else is in your mind beyond the words ' you have breast cancer.' *This is a shock to your system but we need time to process as you would any life impacting event but would not want to be 'pushed' into counselling - might be relevant on down the line |  |
| **B165.3** | I understand once being diagnosed that what can come next can depend on different outcome, however being informed and learning to deal with treatment on a day by day basis was difficult foe me as I was an organised professional person and my diary had to be handed over to the medical team |  |
| **A169.1** | How do you assess the impact of the diagnosis of breast cancer? Do you recognise that sometimes the reaction is delayed...I went into a kind of bubble that didn't let me feel the impact and people thought I was very 'up' about it. |  |
| **B48.7** | Why isn’t counselling offered as part of treatment plan? |  |
| **A129.6** | Q. Why is counselling only offered years later not at the start. |  |
| **A154.1** | I would have preferred to have been told the complete story of my diagnosis rather than part of it because they didn't want me to wait any longer |  |
| **A159.1** | Identifying the best method for each person to receive their diagnosis/outcome of tests, waiting for results is one of the hardest things to deal with, having some control over how that is given would be welcome. Providing patients with medical reports and test results if required - some people don't know they are able to request these. |  |
| **A161.1** | What kind of emotional support should be given at time of diagnosis? What is the optimum time for tests to be carried out and what can be done to speed the process and get timely results? How should the order of treatment be prioritised ie surgery before chemo, or the other way round |  |
| **A170.5** | What is the best information to provide at the time of diagnosis? |  |
| **A80.1** | Support at the consultation phase for counselling referral. |  |
| **A52.1** | From first mammogram to double mastectomy was 9 weeks No counselling was offered I had to ask and do my research through support group on Facebook |  |
| **A49.3** | Do you think the timeline between testing, diagnosis and communication of a diagnosis of cancer is reasonable? How do think the person experiences the waiting time between testing and diagnosis? |  |
| **A124.1** | Finding more support on a one to one basis at the time of diagnosis, with gentle explanation of what lies ahead of treatment. |  |
| **A142.1** | How to best support people at the time of their breast cancer diagnosis? How helpful would written literature be at the time of diagnosis. (Patients have so many questions, but often unable to vocalise these at the time of diagnosis - many of these questions will be similar for all) |  |
| **A110.6** | How can we best support people at the time of their breast cancer diagnosis? This could be included in a written treatment plan. as suggested above. |  |
| **A74.3** | I think that given waiting times, referrals for counselling should be made at point of diagnosis. Most patients will need some emotional support at some stage and this should be more readily available. |  |
| **A84.1** | How can the time from biopsy to diagnosis be reduced? The most stressful time is the period of uncertainty, once you know you can start digesting and coping with the news. |  |
| **A161.1** | What kind of emotional support should be given at time of diagnosis? What is the optimum time for tests to be carried out and what can be done to speed the process and get timely results? How should the order of treatment be prioritised ie surgery before chemo, or the other way round |  |
| **A170.5** | What is the best information to provide at the time of diagnosis? |  |
| **A80.1** | Support at the consultation phase for counselling referral. |  |
| **A52.1** | From first mammogram to double mastectomy was 9 weeks No counselling was offered I had to ask and do my research through support group on Facebook |  |
| **A49.3** | Do you think the timeline between testing, diagnosis and communication of a diagnosis of cancer is reasonable? How do think the person experiences the waiting time between testing and diagnosis? |  |
| **A124.1** | Finding more support on a one to one basis at the time of diagnosis, with gentle explanation of what lies ahead of treatment. |  |
| **A142.1** | How to best support people at the time of their breast cancer diagnosis? How helpful would written literature be at the time of diagnosis. (Patients have so many questions, but often unable to vocalise these at the time of diagnosis - many of these questions will be similar for all) |  |
| **A110.6** | How can we best support people at the time of their breast cancer diagnosis? This could be included in a written treatment plan. as suggested above. |  |
| **A74.3** | I think that given waiting times, referrals for counselling should be made at point of diagnosis. Most patients will need some emotional support at some stage and this should be more readily available. |  |
| **A84.1** | How can the time from biopsy to diagnosis be reduced? The most stressful time is the period of uncertainty, once you know you can start digesting and coping with the news. |  |
| **A137.1** | I had biopsy and titanium marker after mammogram and ultrasound. Afterwards I was put in a room with a book all about cancer. I was almost paralysed with fear. When doctor came in “ can’t tell you for certain till we get results” I was advised. So two weeks of stomach churning waiting. Why did they say - yes it looks like cancer but we’ll confirm it. Two weeks of hell I would rather have told and had a chance to get my head around it. |  |
| **A161.2** | What is the optimum time for tests to be carried out and what can be done to speed the process and get timely results? |  |
| **A23.1** | My diagnosis was a shock, to be told then left alone for a week was hard. Your mind goes into overdrive. Any information I sourced myself on the internet. |  |
| **A29.2** | Providing better information about why tests are being done and what the implications may be |  |
| **A73.1** | It was important to be given full explanations from diagnosis and allowed plenty of time to ask questions in an unpressured setting. A structured programme of treatment with at least provisional dates as removing the lump was very important to me. I needed the support of a time structure. |  |
| **B125.3** | It would also have been helpful to know the optimal timescale for chemotherapy and radiotherapy following surgery, and the impact of infection or other complications. |  |
| **A170.3** | How soon should treatment/surgery commence? |  |
| **A176.1** | Order and timeliness of tests |  |
| **B73.3** | Main question after diagnosis was how soon surgery could be done. |  |
| **A147.1** | Sadly the way I was informed of my breast cancer was handled very badly, I had just had biopsies taken and as the nurse was patching me up I asked what would happen next. The doctor said "Your oncologist and surgeon will discuss a treatment programme", then just walked out of the room, this is how I found out I had cancer! I was then left on my own in a waiting room for 40 minutes until I saw a nurse to make my next appointment. Once I was home I wrote a letter to the clinic about my experience and the doctor concerned was sent for further training. Also a system was introduced where if a patient was on their own they would be given a chaperone if the news was bad, just to sit with them, or talk if they needed it. I was never really given a treatment plan, it was just a rush of appointments with various people for various things. It would have really helped me to know what was going to happen when and why. I was also left to book my own CT/MRI scans and the stress of that was quite bad, trying to get through and then being told no appointments for weeks. I think more information, a rough time frame and reasons for each thing would help to deal with what is going on, and support for those receiving bad news as well. |  |
| **C21.3** | Follow up and support is critical. In my case this was a constant failure. It took four attempts and a letter from my GP before I got sight of my Pathology report. If I had a question this had to be by email and a response would take days. No one called to see how I was. I got far better support from the Breast Cancer Someone like Me service. |  |
| **B116.2** | Again, a plan neds to be formed ASAP. Waiting is part of cancer but no one of us get used to it, the stress associated with waiting is evil. Some explanation are never given by the nurses or oncologists, such as the name of more efficient medicine, info about ports, refund for transportation etc...If you're not actively researching yourself, you can miss out on a lot of help. |  |
| **A126.1** | Please provide much more counselling and information for those with triple negative apocrine cancer. There is a desperate need as there is very little information to be found apart from the morbid, horror-inciting websites. Please publish reliable studies and point sufferers in the direction. | How can we provide better information about less common forms of breast cancer (e.g. lobular cancers) including how this may affect breast cancer surgery and other treatments? |
| **A45.2** | If there was any support for their partner or family? | What is the best way to support the partners/families of patients diagnosed with breast cancer? |
| **A112.2** | What is the best way to support partners/carers and advise them on how to support the patient. |  |
| **A145.1** | Could there be more support for family members, especially under 18s? Two of my children were 12 and 14 at the time of diagnosis. They now suffer from anxiety and depression which they think relates to my having had cancer. My family has a high incidence of breast cancer on my mum's side, although no genetic link has been traced. I have two daughters who are very concerned about their future in terms of developing breast cancer themselves. I think Girls in this position would benefit from some form of counselling/intervention to make them aware of what it means form them in terms of the timing of future breast screening. Also, they need to be taught about checking their breasts regularly. I have done this, but I think they would benefit more if it was part of a wider support package. |  |
| **C155.3** | A buddy support system for after care like emotional and cognitive impairment | What is the value of a ‘buddy system’ for patients newly diagnosed with breast cancer? |
| **C140.12** | support and patient groups which might be a source of comparative information to understand whether the experience is typical or not (and how much to worry) | What is the role of charities and support groups for patients with breast cancer and when are they most useful? |
| **C25.5** | Given information on support groups but especially BreastCancerNow Someonelikeme when you can talk to someone who had your surgery or treatment or how you coped with children etc. |  |
| **A35.1** | Benefits of signposting patients to groups/charities to create support/information networks early on. |  |
| **A2.4** | Should charities such as Macmillan play a role immediately after diagnosis particularly for patients from low income families? |  |
| **A86.1** | How useful are support groups and on - line communities in giving up to date information and support? |  |
| **A48.4** | Why aren’t more charities/support groups widely advertised? We need more than just our designated nurse. |  |
| **C77.5** | There is nothing better than speaking to someone who has been through the same thing. I know everyone's experience is different but speaking to another woman who has had the same surgery is so helpful. Doctors and nurses can only tell you so much. I think having peer support is great, this is something that could be set up? Having a group of volunteers who have previously had surgery, who a patient could call for support and to discuss their feelings and experience. I know that Action Cancer already do this but it only happens after surgery, so giving a patient the option of this from diagnosis would be great. |  |
| **A120.3** | Are counsellors or support groups available for all breathing cancer patients? |  |
| **A86.2** | When is the best time to join in with such (1) groups? |  |
| **A152.1** | I would definitely have benefitted from speaking to someone like myself. I am 4.5 years post treatment and I speak very frankly about it in a positive manner. I had good experiences and I share that. I would have found it useful to know what you feel like physically and mentally after treatment - surgery etc. |  |
| **C160.3** | Access to patients who have already had successful surgery should be offered. They know what to expect and can offer practical support and reassurance. |  |
| **A48.2** | Why aren’t there more younger women support groups? |  |
| **A48.3** | Why aren’t younger women used in campaign/information brochures? |  |
| **A120.1** | Are all breast cancer patients offered a named nurse they can consult at any time? | How can we improve the support offered by specialist breast cancer nurses? |
| **A165.1** | I think it is very important from day 1 of diagnosis that the patient is appointed a Macmillan nurse - or professional individual to go through what they have been told. I was and found it so reassuring. |  |
| **A64.1** | Thinking about my breast cancer experience, do you have any plans to ensure that a Breast Cancer Nurse is genuinely available at reasonable times to answer questions or give reassurance? I was treated at The Cromwell and then The Royal Marsden - two hospitals that announce they are always there for the patient, etc. etc. but I can assure you they were not! And I desperately needed someone to help me join-up the treatments I was having |  |
| **A163.1** | I do believe that contact with someone like BCN should be offered before surgery, not just leaflets or pamphlets. Speaking to someone with experience of breast cancer surgery, on reflection, would have been invaluable |  |
| **A84.2** | How much support from CNSs is required at the different stages of the disease and when do patients need support most? In my experience, the screening centre was full of CNSs, but once diagnosed, it was a struggle to get in touch with anybody for support. |  |
| **A56.2** | And questions as to the impact for those not only without partners, but without close family support, perhaps because they don’t have surviving relatives, or children, or family are separated by distance. Even getting to hospital for surgery and treatment can be difficult. Questions need to be asked around the impact on people who have little practical support in their lives as well as limited or no emotional support. Ask if outcomes of treatment differ for people who have limited access to that kind of basic support, which in my experience is always assumed to be in place by the professionals. | How can we best support patients without a support network who are diagnosed with breast cancer |
| **B2.8** | Are patients with learning disabilities treated differently to other patients regarding options? | Are patients with learning disabilities treated differently to other patients regarding options? |
| **A84.3** | How can communication be improved between patient/carer and clinical team? Would e.g. the use of email be acceptable to clinicians? Would services be more efficient if patients carried their own notes (as well)? I have experienced so many delays due to notes going astray is a source of frustration for patients and clinicians alike. | How can we improve communication between patients with breast cancer and the clinical team? |
| **C168.5** | What psychological support does the cancer patient need - and when do they need it? I am just coming to the end of one year of treatment and I know the psychological impacts will hit soon, but I have no idea where to go to for support. | What psychological or emotional support do patients having breast cancer surgery need? When is it needed and how can we improve the way that it is provided so that more patients can benefit? |
| **A2.2** | Should psychological support be offered throughout diagnosis and treatment? |  |
| **A110.2** | Do people actively referred for counselling have better long term outcomes esp mental health? |  |
| **C169.5** | Increasing availability of psychological support and getting research on the positive impact of psychological services. |  |
| **A92.1** | It was very difficult to go through the diagnostic process without anyone for support during the COVID crisis |  |
| **C49.11** | What psychological support do you think a patient might need post surgery? |  |
| **B90.12** | Can patient support be improved with a combined, co-ordinated support programme from a CNS and former patient(s)? |  |
| **B56.6** | Where can patients turn to get psychological support with their choices if their health trust does not provide an oncology psychologist. |  |
| **C49.12** | Do you think that your local health board provides adequate emotional support to patients undergoing breast cancer surgery in the long term? |  |
| **A77.3** | Counselling is a great help and should be offered at all stages of the cancer journey. |  |
| **A29.1** | Getting the right counselling / support at the right time |  |
| **A140.5** | where they can get counselling, support and information |  |
| **A110.1** | What are the best methods of counselling for people diagnosed with breast cancer? |  |
| **THEME 2: NEOADJUVANT CHEMOTHERAPY AND SEQUENCING OF TREATMENTS** | | |
| **B78.4** | *theatre time / recovery time / possible long term consequences of your decision / what does the timing mean in relation to my survival - what does the evidence show - should I have surgery before chemo / after chemo - being alive is the main concern at this point | What information and support do patients with breast cancer want to help them make decisions about whether to have chemotherapy before surgery or surgery first? |
| **A170.1** | What is the best order of treatments for people diagnosed with breast cancer? What are the advantages/disadvantages of the |  |
| **B169.2** | Is there enough patient choice. I think that some people feel they have to get on the conveyor belt of treatment and don't question it. They might go through chemo before surgery because that is what they have been told, whereas psychologically they might want surgery first to 'get rid of it'. |  |
| **A138.1** | Options? I was only given an option of CHEMO first then surgery because I’d read elsewhere this was possible. Ensure patients can if want have options? |  |
| **B44.2** | I was scheduled for chemo first. I was terrified. All you see on tv is women losing their hair. You don't hear that when it happens your scalp is burning and you are relieved to get rid of it and it shows chemo is working. Mr x chose chemo first for me. I didn't understand or question it but it got rid of my cancer prior to surgery. It was the best choice ever! And I appreciate it so much. It saved my life. I got reduction and reconstruction at same time and mentally this helped so much. |  |
| **B147.2** | Again I wasn't told why it was decided that my surgery would be after my chemo, it wasn't until I went for my chemo sessions and they kept asking which side I had had surgery did I realise that most people have surgery first. I elected to have a double mastectomy due to my breast size and had to put forward a case for this which luckily was accepted. Due to the nature of my cancer (Inflammatory) and the size of the tumour there really wasn't much option about type of surgery as it was a case of just getting all of it out if they could. Reconstruction was not discussed. |  |
| **A94.1** | Choices about neoadjuvant chemotherapy vs surgery. |  |
| **B144.2** | What is the advantage to having surgery before or after chemotherapy? |  |
| **B94.3** | When to move to surgery when neoadiuvant chemotherapy is not tolerated. |  |
| **B140.8** | timing in relation to other treatments |  |
| **A167.1** | The choice of whether to go straight to surgery or have chemo and/or RTh first |  |
| **B118.2** | Yes agree discussion & explanation about when surgery will happen in relation to chemotherapy & why. |  |
| **A123.4** | Which to do first: chemo or surgery; does this choice affect outcome? |  |
| **A161.3** | How should the order of treatment be prioritised ie surgery before chemo, or the other way round |  |
| **B170.8** | When should chemotherapy be given before breast cancer surgery? |  |
| **B110.9** | What is the best order of treatments for people diagnosed with breast cancer? What order gives the best outcomes? |  |
| **A74.6** | For patients starting neo-adjuvent chemo, they tend to move to oncology and therefore do not have access to a breast care nurse which seems to fall under the surgery side of things which i think can be very isolating for the patient as they aren't able to build up a rapport and this can also lead to a delay in referral or signposting to support services. | How can we best provide support for patients having chemotherapy before surgery during their treatment? |
| **A54.1** | For patients undergoing neo adjunctive chemo more support needed for the pre surgical period. |  |
| **B112.5** | Best order of treatment for successful outcome - Pros and cons to different orders of treatment Best order of treatment for successful reconstruction | Best sequencing of treatments for patients wishing to have breast reconstruction? |
| **B170.9** | Should radiotherapy be given before breast cancer surgery? | Should radiotherapy be given before breast cancer surgery? |
| **A126.1** | Please provide much more counselling and information for those with triple negative apocrine cancer. There is a desperate need as there is very little information to be found apart from the morbid, horror-inciting websites. Please publish reliable studies and point sufferers in the direction. | How can we provide better information about less common forms of breast cancer (e.g. lobular cancers) including how this may affect breast cancer surgery and other treatments? |
| **THEME 4: BREAST RECONSTRUCTION** | | |
| **B53.3** | Why are some people told that if they do not have immediate reconstruction at the time of a mastectomy they will not be allowed it in the future? I was told this, it was not the right time for me to have reconstruction due to being a carer to a disabled child and now have to live with only one breast for the rest of my life. Why are some people allowed delayed reconstruction and others are not? | Why is there variation in whether (or not) women are offered breast reconstruction surgery? How can we ensure that all patients have equal access to appropriate breast reconstruction options, either at the time of surgery or at a later date? |
| **B25.2** | Reconstruction can be done immediately during Mastectomy but not in a lot of cases so this should be explained |  |
| **B143.2** | Surgeons may have a speciality in terms of reconstruction and could inadvertently recommend their preference or specialism rather than being more objective with the patient about their options. I think consideration and the discussion of the longer term impact of surgery prior to it happening would be useful. |  |
| **B146.2** | As above immediate reconstruction made a big difference. Could have had more info on downsides eg time in hospital or breast would go hard with radio - was told this with radio I think. Would not have changed my decision. Was so grateful to my wonderful surgeon and the medical team. I now have one normal bit droopy boob and one Victoria Beckham I say but that’s over 10 years on. Different women have different views but it’s really important that they get info they need and not the politically correct. There seems to be a view that feminists should be flat get a tattoo etc I’m a proud feminist but don’t agree |  |
| **B113.3** | Some surgeons say no reconstruction at time of mastectomy, others do at the same time - why? |  |
| **B58.2** | Mine was full mastectomy with delayed reconstruction. After adjunctive chemotherapy. I think this helped as I went into the chemo strong and had time to get my head around the fact I would have a mastectomy. I still haven’t had reconstruction 4.5 years out through queues and COVID. Now this isn’t an option as have developed secondary BC. I have requested elective contralateral mastectomy. Am hoping I can once stable. | How can we reduce waiting lists and improve access to delayed breast reconstruction for patients who had mastectomy for breast cancer? |
| **B114.2** | Is the choice of delaying reconstruction and the effect on recovery being investigated? | How do the outcomes of breast reconstruction performed at the time of mastectomy compare with those of breast reconstruction performed at a later date? How do both options affect the quality of life and well-being of patients with breast cancer? |
| **B56.3** | Whether immediate or delayed reconstruction is better in each individual circumstance. |  |
| **B170.10** | When reconstruction is due before chemotherapy, should surgeons anticipate weight gain due to steroids/ changes in body shape due to chemotherapy |  |
| **B168.4** | What factors need to be taken into consideration, to give the patient a full understanding of the impact of having a delayed reconstruction versus immediate reconstruction. What are all the surgical options and what are the pros and cons of each? Is surgery required immediately? |  |
| **B153.3** | Do people with reconstruction at time of / shortly after mastectomy have better mental health outcomes |  |
| **B93.2** | Reconstruction should not be delayed as this will cause more psychological problems. |  |
| **A167.2** | To have an immediate reconstruction seems rash because patients are still coming to terms with the whole issue |  |
| **B78.3** | *as above - clear photos of what is involved in the surgery and in reality what you can expect your body to look like from reconstruction or no reconstruction | How can we ensure all patients are given fair and balanced information about all types of breast reconstruction including why some may not be suitable? What information do patients want and how should this be provided? |
| **B113.4** | Some say you have to lose weight first {before reconstruction}? |  |
| **B90.13** | Does a patient's lifestyle affect the type of surgery and/or reconstruction they choose to have and, if so, what lifestyle aspects in particular? |  |
| **C24.5** | Guidance on reconstruction choices (eg I had an immediate implant reconstruction, and mainly was told about how it would look - not how it would feel). |  |
| **B161.6** | How should the best option for reconstruction be discussed and decided? |  |
| **B169.3** | Is there enough information given about reconstruction...the fact that it is not a new breast and that it can go horribly wrong. Is it just the fashion. |  |
| **B155.2** | Reconstruction surgeries and types explained. Perhaps patients who have had are asked if they can share experience.... pro/cons before decisions are made. |  |
| **B13.3** | What impact on successful reconstruction does radiotherapy have? | How does radiotherapy affect the outcomes of breast reconstruction surgery? |
| **C90.23** | Capsular Contracture: What is the best method of identifying the cause of a patient's capsular contracture? | How can we reduce the risk of patients developing problems after implant-based breast reconstruction in the short and long term? |
| **C90.24** | How can the incidences and/or severity of capsular contracture be reduced during and/or post-surgery? |  |
| **C90.25** | Implant Movement / Rotation : What are the best methods to reduce implant movement and/or rotation? |  |
| **B90.11** | Can the feel (not just the look) of different reconstructions, particularly implant reconstructions, be simulated? | Would images including 3D scanning or computer simulations showing the likely cosmetic outcomes of different operations help patients make better decisions and improve their satisfaction with breast cancer surgery? |
| **A6.1** | Does having access to surgical photographs in the clinic help manage patient expectations over surgical outcomes or decisions over which surgery to choose? |  |
| **B6.2** | Would 3D surface scanning of breast size/shape prior to surgery help with reconstructing a closer matching breast shape? |  |
| **B6.3** | Would giving patients the option to have 3D breast scanning prior to surgery in order to have that data stored and available for making better matching prostheses help with self confidence after surgery? |  |
| **A127.2** | Would it be possible to develop a computer graphic of what a breast would look like after surgery so that people who are advised they will get a cosmetically poor result from a lumpectomy can make an informed decision on if it is the best option for them? This would also help patients know what to expect. |  |
| **B110.12** | What is the best type of breast reconstruction to have for longevity? | What are the short and long-term outcomes of different types of breast reconstruction operation and how do they affect patient’s quality of life and well-being? |
| **B55.2** | What are the risks in relation to reconstruction - how many lead to further surgery required. |  |
| **B153.2** | Understanding the longer term effects of the surgery options - what will result in longer term success (ie not need replacing again if reconstructed) |  |
| **B112.7** | Best treatment for reconstruction |  |
| **B120.4** | What is the success rate of breast reconstruction done at the same time as the breast surgery? |  |
| **B170.7** | What is the best type of breast reconstruction to have? |  |
| **A30.2** | Breast reconstruction - how do synthetic and natural materials compare for those patients wanting breast reconstruction? |  |
| **B159.3** | More work and honesty about immediate and long term surgical outcomes. The differences between a 'successful' outcome for the surgeon compared to the patient, e.g. at the time, my reconstruction was a 'success' as the operation went well, the result was good (initially). However it was the most painful thing I've ever endured (more than induced childbirth without pain relief) and the final outcome is a disaster, added to which, my back is permanently damaged (LD reconstruction). Pre-op the surgeon said that 'some' women found it uncomfortable, most don't notice the difference. I wonder if this has ever been properly researched - my surgeon's definition of success is completely opposite to mine. I have massive regrets about undergoing the operation, looking back, it's madness to destroy one functional part of the body for a purely cosmetic result. |  |
| **A117.2** | Is one reconstruction method preferred over another and if so, why? |  |
| **B110.10** | I What are the long term complications and impact on general health & quality of life post reconstruction? |  |
| **B110.13** | What are the long term risks for those who have a breast implant along with reconstruction & would follow up reduce risks |  |
| **B110.14** | what is the shelf life of implants, do they need replacing after so many years? |  |
| **B148.2** | What are the end results, survival, cosmetic, sensation, of the different options |  |
| **A117.1** | Can I have reconstruction/when/what kind and how will this effect my treatment plan? | Does breast reconstruction surgery affect future cancer treatments or long-term cancer outcomes? |
| **B171.4** | will surgery delay my radiotherapy? |  |
| **B24.3** | Specifics regarding timing and impact of further treatment (eg if having an implant, potential complications for radiotherapy). |  |
| **A153.1** | How different surgeries make a difference to long term outcomes |  |
| **B139.1** | For patients (likely in 20's, 30's) who have had mastectomy with delayed reconstruction, but are not suitable for implant based reconstruction and don't have much 'spare' tissue for autologous reconstruction, is there research into alternative suggestions for these patients? e.g. growing tissue in the lab, expanders used elsewhere on body, improved fat grafting, scaffold mesh to model breast shape etc? | Are there any alternatives to implant-based reconstruction for women who do not have enough tissue for a tissue-based reconstruction? |
| **B114.1** | Is it always necessary to remove the nipple with mastectomy? I had Latissimus Dorsi reconstruction at the same time as my mastectomies. I am so glad I had this. I would not have wanted further surgeries. | Is nipple sparing mastectomy a safe option for patients with breast cancer having breast reconstruction? |
| **THEME 3: BREAST CANCER SURGERY** | | |
| **C35.6** | Short term and long term outcomes on social/emotional well-being in relation to all mastectomy outcomes. | What are the short and long-term outcomes of mastectomy surgery and how does mastectomy affect patients’ quality of life? |
| **A112.4** | Best type of operation to avoid recurrence. | What are the short and long-term cancer related outcomes after breast conserving surgery and radiotherapy vs mastectomy? Which operation reduces the risk of recurrence most? |
| **B86.3** | What is the risk of recurrence following a mastectomy versus a lumpectomy? |  |
| **A39.1** | What level of recurrence is there of breast cancer for partial breast removal vs full mastectomy? |  |
| **B38.3** | Why do practices vary so much between health authorities? | Why is there variation in the types of breast cancer treatment offered in different areas of the UK?  (not just types – why is there variation in breast cancer treatments and outcomes in the UK and how can this be addressed) |
| **A51.1** | Why is treatment a postcode lottery in Northern Ireland? There are huge differences in the type of treatments, drugs offered and waiting times for surgery depending on which Trust/hospital a patient goes to. Patients don't realise this unless they do their own research. |  |
| **B51.3** | Again depending on which Trust you attend in NI the information on choice and timing of surgery is of varying detail. |  |
| **B133.2** | There seems to be a postcode lottery in the UK as to what is offered and when . There needs to be a system offered that is set in stone |  |
| **B2.6** | Are these questions {ask to patients to determine choice & timing of surgery} consistent across oncology centres? |  |
| **A118.1** | Counselling & support re different surgery options including reconstruction. I had reconstructive surgery for breast cancer but following year it was not offered or discussed with my 74year old mum. Important to have discussion | How can we ensure that all patients with breast cancer are offered and given fair and balanced information about all appropriate types of breast cancer surgery? How can we best support patients to make decide what option is best for them? |
| **A140.6** | information that takes account of their personal circumstances |  |
| **B53.4** | What types of surgery options for cancer removal are there? ( I was given no choice in the type of cancer removal surgery and no explanation as to why that was the best option) |  |
| **B142.2** | Advantages and disadvantages of different types of operations for breast cancer? |  |
| **B24.2** | Information on the different types of surgery and the impact of that. |  |
| **B30.5** | What is the right breast surgery for the individual patient? Avoiding over, under treating. |  |
| **B179.2** | How might my breast(s) look after different types of surgery? |  |
| **A155.1** | I would like to know my choices of surgery and explained in clear, simple and concise conversations. |  |
| **B74.7** | I think that patients should be given information about their surgery decision in relation to the surgery options that are available. At diagnosis, patients can assume that they will automatically have a mastectomy from their knowledge of breast cancer in the media. I think that each surgery option should be explained and why a particular surgery is best for you. I think that patients need to understand better why certain surgery decisions are being made. I think that information about risk also needs to be made clear to patients when discussing surgery decisions. |  |
| **B142.3** | Which types of surgery are suitable/possible for which patients? |  |
| **B77.4** | I think patients will always be guided by surgeons and breast care nurses. When you are diagnosed with cancer your first thought is " am I going to die" The choices for surgery can be overwhelming and not something that you really want to have to think about, so clear guidance from professionals is essential. You have to trust that your team will always recommend what is best for you based on your own situation. |  |
| **B140.7** | pros and cons of different types of options (including information that allows the individual to consider the implications of their age and other circumstances) |  |
| **B148.2** | What are the end results, survival, cosmetic, sensation, of the different options |  |
| **B170.6** | What are the advantages and disadvantages of different types of operations for breast cancer? |  |
| **B92.2** | I was informed of all the options for surgery and dates although with hindsight I would have liked more detail on the options. |  |
| **A113.1** | Options more clearly laid out |  |
| **A76.1** | My original diagnosis at Kings was unnecessarily alarming: the locum breast surgeon was eager to do a double mastectomy. In the end, with Prof Vaidya, I had only a small lumpectomy. I was lucky. But it could have gone the other way and I would have endured a huge procedure FOR NO REAL REASON. I remain convinced that the man wanted to practice his skills and did not care too much about the patient. I now think I should have made a formal complaint but at the time there was a lot going on. |  |
| **B125.2** | Certainly different surgical options. I didn't want reconstruction but maight have done for all my clinicians knew - it was never discussed. |  |
| **B175.2** | As above - plenty of time to choose option. Impartiality of surgeons. The patient needs the best option for her, not the surgeons favoured procedure. Need good unbiased information. |  |
| **A25.1** | I believe every person should be offered All surgery and advised that their consultant may only perform certain surgeries but given all information ie how long operation will take, recovery time, how long unable to drive etc so that the patient can determine if they want a quicker recovery etc. More information and pictures and not of old people there should be differing age groups so that you can make informed decision. Allow breast care nurse to answer questions on any of above |  |
| **C175.3** | Clear information and assurance. Be honest. |  |
| **B154.2** | Was shocked to be told breast being removed in 3 days. There was no discussion about reconstruction |  |
| **B37.3** | Does the surgeon have a preference to a certain type of surgery? Is he/she prepared to listen carefully to a patients choice and act on it? Even if it goes against what they would usually recommend? |  |
| **B80.3** | The options need to be given to the patient - all the different techniques available apart from the specific consultants expertise. |  |
| **B149.2** | I found it appalling my surgeon only offered me 2 options and what I hadn’t realised is that she could not do the other operations. If an operation is so expensive it limits everyone being offered surgery is that fair? |  |
| **A13.1** | What measures or audits are in place to ensure that newly diagnosed patients are routinely offered ALL available information about their surgical options and not just the bias of a particular surgeon or centre (particularly with reference to aesthetic flat closure and/or prophylactic contralateral mastectomy). |  |
| **B37.2** | What options for surgery do I have? Options should include aesthetic flat closure; no reconstruction now or ever. |  |
| **B56.4** | Whether reconstruction options are right for each patient. Does the patient have a choice? Why is reconstruction always discussed, but staying flat rarely mentioned? |  |
| **B21.2** | This is also crucial. I had a lumpectomy but the option of reconstruction at the time of the operation was never mentioned so I ended up with uneven breasts. When I raised it later I was told I would have to go privately. | How can we ensure that patients are offered appropriate and equitable access to oncoplastic breast conserving surgery including symmetrisation (balancing surgery) if needed? |
| **B151.2** | I did not fully understand why the surgeon was not doing his best to make the breast symmetry look the best post op at the time of doing the surgery and why this was proposed as reconstruction. They risks of another op with lymph nodes removed felt to gréât to have any reconstruction done. |  |
| **B23.2** | I have a mammoplasty and have dropped at least two cup sizes. At no point was anything mentioned regarding having a reconstruction or an implant to level things up |  |
| **A166.1** | Being able to determine the need for actual surgery (i.e. cancer) vs exploratory surgery (unknown, suspected) vs ensuring clear margins during first surgery to avoid a repeat visit to theatre (lumpectomy) | Is it possible to avoid the need for further surgery in patients having breast conservation procedures where not all the cancer is completely removed? |
| **B52.2** | I was dazed when told I had to have mastectomy handed two spongy implants numbers on them ....why can’t they be sized like bras A,B, C , D , DD . | How can we make prostheses better and easier to use after mastectomy for breast cancer? |
| **THEME 5: CONTRALATERAL MASTECTOMY AND FLAT SYMMETRY** | | |
| **B161.5** | When should mastectomy be considered for psychological reasons, for example, removing a healthy breast? | Are there situations when the removal of the healthy opposite breast should be considered for psychological reasons in patients with breast cancer who have had a mastectomy but do not carry a breast cancer gene? |
| **B35.5** | Lack of symmetry surgery availability for those not wanting reconstruction (CPM). | Why is there variation in whether women who do not carry a breast cancer gene are offered a balancing mastectomy as an alternative to breast reconstruction after a single mastectomy for breast cancer and how can this be addressed? |
| **B34.2** | To be told if it did come to a mastectomy and you can't get reconstruction they will not remove the other large breast but reduce it why ? Once I knew that I needed a mastectomy and the thought of living with one breast nearly destroyed me. |  |
| **B36.4** | Why is symmetrical flatness not talked about in this section? |  |
| **B49.9** | Is it reasonable to discuss a double mastectomy if the patient requests that but only one breast has cancer? |  |
| **A57.1** | Why are women not offered double mastectomy when a single mastectomy is the necessity? |  |
| **A38.1** | Why do Breast Units presume reconstruction is the norm? Why is no reconstruction not presented as an option? |  |
| **B29.3** | Understanding why many surgeons are not happy to allow or do not understand why many women would want to live flat after breast surgery (ie no reconstruction) | How can we support healthcare professionals to routinely offer balancing mastectomy for symmetry as an alternative to breast reconstruction in patients who do not carry a breast cancer gene who have had a single mastectomy for breast cancer? |
| **A48.1** | How can we be better supported to have our decision accepted to want to have a double mastectomy for symmetry? Why is this never an option when having an implant or tissue reconstruction is a longer recovery and comes with more risk abs complications? |  |
| **B49.7** | Do you agree that the option to stay flat is a legitimate one and would you support your patient's choice without reservation? |  |
| **A57.3** | Why is it not understood by surgeons that living with one breast is a peculiar situation? |  |
| **B90.14** | Bilateral mastectomy: How many women (with no BRCA gene mutation) Are offered a bilateral mastectomy as a surgical option at diagnosis, alongside all the other options? |  |
| **B49.5** | When you talk to a patient who has been diagnosed with breast cancer that requires a mastectomy do you discuss all options with them from the outset? |  |
| **B35.5** | Lack of symmetry surgery availability for those not wanting reconstruction (CPM). |  |
| **B13.5** | How many centres and what are the patient numbers who offer contralateral prophylactic mastectomy as a real and viable alternative to reconstruction? |  |
| **C57.5** | Why reconstructive surgery is offered (pushed) but symmetry surgery is not even discussed. |  |
| **A57.2** | Why are women offered reconstruction but never a symmetry mastectomy? |  |
| **A39.3** | If staying flat post-mastectomy is presented as a valid choice with equal validity to reconstruction, what effect does this have on the proportion of women choosing to have/forego reconstruction? |  |
| **B171.3** | What options do I have apart from reconstruction? |  |
| **B56.5** | What support is available for patients choosing to stay flat? | How can we best support patients who chose to stay flat after mastectomy for breast cancer? |
| **A123.5** | Is there a way, or ways, of helping women still feel feminine after a double mastectomy? Do these women benefit from specialist counselling? |  |
| **A113.2** | Emotional support for post mastectomy |  |
| **B60.1** | I kept getting asked if I was sure I didn’t want reconstruction and that I might change my mind and felt that because I didn’t want reconstruction I was just seen by training drs |  |
| **A39.2** | Breast cancer treatment really pushes post-mastectomy reconstruction. |  |
| **B35.4** | Level of discrimination against women not wishing to have reconstruction. |  |
| **A36.3** | Why aren’t ladies offered the chance of flat symmetry at the time of diagnosis. | In patients having mastectomy for breast cancer who do not carry a breast cancer gene, should a balancing mastectomy be offered at the same time or at a later date? What factors may influence this decision? |
| **B61.5** | Why cant I have both breasts removed together |  |
| **B90.15** | b. How many women (with no BRCA gene mutation) ask for a bilateral mastectomy (with or without reconstruction) at diagnosis but are not offered it as an option or are discouraged from it? |  |
| **B90.16** | How many women (with no BRCA gene mutation) are offered and/or ask to have a bilateral mastectomy and choose to have it? |  |
| **A35.2** | Risks/benefits of timing of CPM when requested by patient at same time of single mastectomy (eg at same time or months after a single mastectomy) |  |
| **B161.4** | Is the approach around surgery correct? Ie should it be considered that reconstruction is the best option? | Are women who have opted for balancing mastectomy for symmetry satisfied with their decision and how do they feel about the results of their surgery? How does this compare to women who opted for breast reconstruction?  How do the outcomes of CSM compare with BR |
| **B13.2** | What comparative studies have been published with regard to outcomes and complications of reconstruction or flat closure? |  |
| **C13.6** | What are the comparative long-term satisfaction statistics, complication data and quality of life analysis of patients offered aesthetic flat closure and/or contralateral prophylactic mastectomy compared to reconstruction? |  |
| **B49.6** | What are the advantages of reconstruction over staying flat? |  |
| **A39.3** | What are the outcomes in terms of satisfaction with treatment for women undergoing reconstruction Vs women choosing to go flat? |  |
| **B90.17** | Of the women who choose to have a bilateral mastectomy, how many are happy with their decision? |  |
| **B90.18** | Of the women who choose to have a bilateral mastectomy, how many regret their decision (including reasons why)? |  |
| **B13.4** | How many centres offer aesthetic flat closure as an option at the time of diagnosis? | What is the best way to perform a mastectomy operation to achieve an aesthetic flat closure? How can we ensure that all surgeons are trained to perform this and can offer it routinely to patients who do not want breast reconstruction? |
| **A94.2** | Discussing different scar possibilities if going flat. |  |
| **B49.8** | If a patient expresses a wish to stay flat do you have the knowledge and skills to ensure that the end result is as neat and tidy as possible or should they be referred on the a plastic surgeon post mastectomy as an integrated part of their treatment? |  |
| **C162.1** | How will you ensure an aesthetic flat closure for those patients who do not wish to undergo reconstruction? |  |
| **A54.2** | More development of aesthetic flat closure for those patients who do not have immediate reconstruction |  |
| **THEME 6: SURGERY AND POSTOPERATIVE CARE** | | |
| **C2.9** | Care should be seamless between hospital and home. | How can we ensure continuity of care between hospital and the community for patients having breast cancer surgery? |
| **C92.3** | I have had no contact/ phone call from my GP/surgery since my operation 6 months ago. The surgeon and support nurse are readily available for a chat or check . |  |
| **C78.5** | *important to know what to expect when you come round after surgery - I expected to be wrapped round in bandages but in reality a simple dressing set on top of 'staples' for my full mastectomy | How can we best prepare patients for breast cancer surgery including aftercare what to expect after the operation? |
| **C78.6** | * important to be ready to look at the area and the change in losing a body part - what makes me me. |  |
| **C80.5** | Also the after care is not discussed. Bras, Prosthetics etc etc are also not even mentioned and the patient is having to ask support groups for help. |  |
| **A24.1** | How best to give patients information about their surgery to take away from the appointment where they are first told they have breast cancer and would be needing surgery. (eg some sort of booklet with tick boxes and links for further support). |  |
| **C138.8** | Research welfare of aftercare kits, canvas bags provided to hold draining bags discreetly, armpit cushions (I was donated these and they gave me some dignity) |  |
| **B24.4** | Detail on the expected recovery time and side effects, and what physio may be needed afterwards. |  |
| **C154.3** | More information on what to expect when I woke after surgery Wish I had known problems with reconstruction |  |
| **C142.4** | Short-term and long-term outcomes. What to expect in terms of recovery, discomfort, ascetics at what stage post surgery |  |
| **C24.6** | Guidance on recovery in hospital - what to take, how long likely to be in hospital. |  |
| **B1.4** | What does the wire do? My experience was that surgery took place quickly after diagnosis. The team were really good & explained things well to me. When discharged after surgery, the team explained that I needed to start doing exercises to enable me to raise my arm & keep it out of the way |  |
| **A65.1** | How can I best prepare for surgery. |  |
| **C73.6** | I wanted to know when I would have the results especially if further surgery was going to be necessary. |  |
| **C137.3** | My operation went fine and healed wonderfully. I would have liked more info and explanation about the effects of having nodes removed. |  |
| **C74.10** | Preparing patients for the potential of further excision for better margins may help patients cope with the prospect of this if that is required. |  |
| **A58.1** | I was fortunate at my health care trust to have a BCN present at time of diagnosis. Plus a very good cancer support centre. I know many others are not as fortunate and on forums ask fairly basic questions such as surgery recovery times, driving, drains. |  |
| **C116.3** | My after surgery was fine, but felt confined to a booklet. WE get lots of booklets, and they can be overwhelming |  |
| **A73.2** | I wanted to know the lasting impact of a lumpectomy, scarring and change of shape to breast. |  |
| **C61.8** | Is breast removal major surgery |  |
| **B73.4** | On the day of surgery it was important to meet the surgeon prior to the operation and go through again exactly what was going to happen. What and how much was going to be removed including lymph nodes. |  |
| **C150.2** | Loss of feeling of such a large area following surgery was not really explained at all Ongoing pain at the tumour site was not expected for so long and was very worrying for a long time after surgery. This could have been explained further. I was told my scar would follow my bra line - it’s nowhere near. I don’t mind but Surgeons should be honest. |  |
| **A150.1** | Information regarding recovery time, rehabilitation and how the area will feel following surgery. Focus at my diagnosis was about conserving the breast but I wasn’t fully aware of how it would feel afterwards, just how it would look which I don’t feel looking back is as important for me. |  |
| **C161.8** | How should outcomes be explained prior to op? |  |
| **C159.5** | Information about ways to make patients more comfortable, e.g. how to obtain items/equipment such as mastectomy pillows, drain bags, etc. |  |
| **C99.2** | What is the rate of winged scapula after mastectomy, and what are the best ways of preparing people for the possibility, treatment options if any, and support? |  |
| **C78.8** | *an understanding that cording might happen and that it is painful but fixable. |  |
| **C55.3** | Is a drain necessary in all cases? | Why is there variation in the use of surgical drains after breast surgery and are they always needed? |
| **C78.7** | *explanation of why your drains are in and what they do. |  |
| **C53.5** | Why do some health trust use drains and others do not. |  |
| **C112.9** | Best form of drain management and pain relief |  |
| **C38.4** | Why do some hospitals use drains and others not? |  |
| **C25.4** | You can be discharged with drain and idea to be given bag for drain Drain dolly to carry them when moving. |  |
| **C149.3** | You can cope with the drains at home and if you are able early discharge is better as your own surroundings are better. Definitely insist on at home removal of drain |  |
| **C113.5** | Preparation for what a chest drain is and how it feels. |  |
| **C131.3** | Drain management |  |
| **C144.3** | How can drains after surgery be more manageable and less painful? |  |
| **C80.4** | My experience is this is all very different - my first ever surgery I was totally not informed about drains - this is something needs to be spelt out pre surgery. |  |
| **C56.7** | The advantages vs disadvantages of having drains rather than pressure dressings. |  |
| **C83.2** | Is there a better way of draining, instead of carrying a little flowery bag . |  |
| **C38.5** | Why do some surgeons use dressings and others don’t? | Why is there variation in the use of dressings, which are best and when should they be removed? |
| **C55.4** | When is best for dressings to be removed? |  |
| **C179.3** | What sort of dressings should I use to protect my wound, and will they be provided? |  |
| **C38.6** | Why is there not a specific ward for women returning from breast surgery? My experience was women after breast surgery were dotted around different wards in the hospital along with patients with varying needs. I was in a bed next to an elderly patient with dementia, not with the women of sat with all day waiting for surgery and talking about our feelings. | How can patients having breast surgery best be managed in hospital after surgery? Do all patients having breast surgery require an overnight stay? |
| **C169.4** | Have you considered that the post-op specialist in-patient NURSING care is paramount. Just being chucked out after major surgery by non-specialist nurses is not good. Have you considered having a breast cancer ward or unit. Practicalities seem well taken care of but TLC in-patient care lacking perhaps. |  |
| **A170.2** | Can patients choose which hospital the surgery will take place at and if they can be guaranteed a side ward/quieter more private bed? I ended up transferring my treatment because I would not have coped on a nightingale style ward. |  |
| **A167.3** | Day surgery seems to me to be utterly inappropriate. A few days in Hospital means all the worry of drains and dressings are coped with in the company of ladies with similar problems. It’s not just physical but emotional and psychological, |  |
| **C29.4** | Better support to be discharged home after surgery | How can we best provide support for patients after breast surgery after discharge from hospital? |
| **C37.5** | What to expect once home, bearing in mind most surgery is day case, is so important to reduce anxiety post-op. |  |
| **C124.3** | Help to find more home support & after care. |  |
| **C93.3** | More follow up appointments needed, rather than being discharged and left to manage on your own. |  |
| **C120.5** | Following discharge after breast reconstruction, how frequently are patients followed up? Are patients fully briefed on what to expect if something goes wrong eg infection? |  |
| **C51.6** | Dressing management could be carried out by district nurses and GP's but they seem reluctant to do this and will often tell patients that they should go back to hospital for this type of thing. There should be a decided nurse in every GP practice who could deal with post surgery cancer patients on dressings, wound management etc |  |
| **C159.6** | Agreed treatments for any complications, I had a small area of infection, called the breast nurses who told me to see the nurse at my GPs surgery and ask for the surgical wounds to be re-dressed. The GP nurse decided that medical honey would be suitable, which did absolutely nothing and the wound worsened. The breast nurses then told me to request medicated dressings by name - if that had been agreed beforehand or I'd been told exactly what should be used, I would have saved myself from yet more pain and discomfort. |  |
| **C117.3** | How can I check for signs of infection post surgery? | What information and support do patients need to detect complications and deal with problems immediately after breast surgery so they can be effectively treated? |
| **C2.11** | A simple flow chart showing who should be contacted if various issues arise would save time and worry for patients. |  |
| **C29.5** | Outcome and side effects of surgery - better preparing people for the possibilities and what the signs and symptoms are |  |
| **C37.4** | All and any potential complication or side effect of surgery must be explained in full, in person with each patient. |  |
| **C53.7** | Why do some people have large bleeds that require emergency surgery within hours after the initial surgery? |  |
| **C53.8** | How can clearer information about complications and side effects be given? |  |
| **C153.4** | Complications and how to pick up and deal with quickly e.g I have permanent nerve damage which means I can’t lift my arm over my head and no one picked this up, it’s only when I noted a few years later to my gp did I have tests to confirm extent of damage |  |
| **C171.5** | How should complications of surgery be communicated and managed (I ended up in hospital on IV antibiotics unnecessarily for 5 days for what was thought to be an infection but turned out to be an allergic reaction). |  |
| **C52.4** | I had to go back to hospital as drain coming out, ended up in a&e on a Friday night three days after operation as my breast cancer ward does not stay open during weekends . Being discharged the same day as your operation with no training of drain change or pain relief, a person did visit on this day from breast care ward but I was too spaced out from operation to take on what she said |  |
| **C84.4** | What are the risk factors for the development of a seroma after breast cancer surgery involving removing lymph nodes; is the amount of drainage liquid straight after operation an indication of likely development and is the development of a seroma linked to the development of lymphoedema? | Information about seromas including when they should be drained and how can be avoided? |
| **C170.13** | How soon can/should you get a seroma drained after developing one? |  |
| **C74.8** | More information about seromas and the potential for cording should be made available for those who have full axillary node clearance. |  |
| **C114.3** | Would it be possible to provide more advanced notice of seroma and drain management to patients following mastectomy and reconstruction?. |  |
| **C170.12** | What is the frequency/risk factor of developing a seroma? |  |
| **C127.4** | Is it possible to predict who will get seromas or hematomas and have the management for these built into the patients treatment plan rather than patients having to rely on A&E and walk in centres to receive help for complication management and dressings? |  |
| **C90.19** | Seroma: What is the best method (during and/or post-surgery) to minimise the amount of seroma? |  |
| **C112.8** | Seromas - what causes them; how to reduce likelihood of occurrence; how best to treat them; when to get extra help. |  |
| **C56.8** | What is a seroma, and his to manage it. Why seromas are not talked about until they appear. |  |
| **C54.5** | More investigation into seromas and how to prevent them. |  |
| **C117.4** | Is it normal to have fluid build up and fluid leaking from the wound in the days post surgery? |  |
| **C33.3** | I recently had no drain after further axilla surgery and this caused 4.5 months of trauma, seroma that was huge 1/2 litre being drained off at a time. Which turned into a hematoma. Because of previous surgery this should have been given more thought. Because it was only a day case "it wasn't necessary" but then the time, cost, effort that i had to go back and forth with problems which ended up in psycological issues were massive and affected me greatly. I feel having a drain in would have eased the issue even if it was for a few weeks. Additionally this was under lockdown so then i had further appointments that meant possible exposure. I've ended up with lymphoedema which wasn't immediate and i personally feel if all this fluid wasn't there i wouldn't have ended up with an additional problem |  |
| **C159.4** | As above. Appropriate and fast pain relief on hand - I was in the worst pain of my life, crying for help, after I came round from surgery and no one seemed to be able to help. Knowledge of who/what to ask for - it was only because I'd cared for a relative that I knew the hospital had a pain team and I asked to see them. Agreed pre-op procedures - the anaesthetist for my op didn't see me the night before and therefore I wasn't allowed any pre-op meds, although before I'd been admitted, this was promised. He brushed off my request for a pain block, saying I wouldn't need it. He apologised afterwards, when he read my post op notes - too late then of course. | Best way to manage pain after breast cancer surgery and  How can we prevent chronic pain after breast cancer surgery? |
| **B129.13** | Q. Why is pain relief not discussed at length, in a manner where doctors can holistically hear the symptoms of pain and calculate the correct medication in the earliest of stages, rather than misdiagnosing causing greater more painful side effects. |  |
| **C25.3** | Careful not to over prescribe painkillers as Tramodol as highly addictive. |  |
| **C23.3** | I was told that breast surgery is one of the less painful surgeries - they lied! I wasn't given any painkillers, just a leaflet and yold to wear a high impact spirts bra 24/7 for at least gour weeks. I couldn't raise my arm and had to get my husband to wash me (including my hair). I couldn't lift my hairbrush. What would happen if i had been living alone? |  |
| **C140.13** | treatment for pain and dressing management |  |
| **C94.5** | Management of immediate post operative pain for people with NSAID intolerance. Extent and prevalence of nerve block training for anaesthetic registrars. |  |
| **C108.6** | Post-operative pain is an issue that is ignored. Lack of follow up leads to a false assumption that chronic pain does not exist in this patient group. It is highly prevalent and causes lots of issues especially relating to QoL and functionality. More research is needed on long term outcomes as well as monitoring of short term outcomes (as we know they are linked). Prevention of or better monitoring of short term pain could prevent persistent pain. Self-management is limited as patients have so little if any follow up and attendance at 'follow-up' programmes is very poor with less than 50% attending these - so how are patients going to effectively self-manage if they do not get this training and information? |  |
| **C44.3** | After my surgery I noticed right side of my stomach looked bit swollen. No one mentioned it. Turned out afterwards it could be lymphedema as it's in my right arm too. I feel I haven't had help with it. I was referred to a different local hospital who wouldn't show me how to do manual lymphedema massage unless I had the 2 week tight wrapping which I refused as I couldn't work with it on. It also takes a while to get used to your new body. | How many women get lymphoedema after breast cancer surgery? Can it be prevented? What is the best way to detect and treat lymphoedema if it develops? |
| **C90.21** | Has the number of cases of breast / chest lymphoedema following breast cancer surgery / treatment increased in recent years and if so, what is the cause of this increase? |  |
| **C90.20** | Lymphoedema: What percentage of patients get lymphoedema as a result of breast cancer surgery / treatment, by type of surgery, extent of surgery, type of reconstruction, and location of the lymphoedema (arm only; arm and breast / chest; or breast / chest only)? |  |
| **C78.10** | * long term impact of lymphoedema |  |
| **C99.5** | Does active monitoring for lymphoedema by clinicians reduce stress (as opposed to patients looking out for, and wondering about, possible signs)? |  |
| **C99.6** | What model of lymphoedema care leads to the best outcomes - emotionally and physically? |  |
| **C99.4** | Does early identification of post-surgical lymphoedema lead to better outcomes? |  |
| **C90.22** | How, surgically, can the risk and/or effect of lymphoedema be reduced, including breast / chest lymphoedema? |  |
| **C30.6** | Best ways for breast surgery to be conducted to avoid lymphedema after breast cancer operation. |  |
| **C151.3** | I guess what worries me most is the lack of knowledge and treatment for lymphodema |  |
| **C65.3** | Better management of nerve damage to the under arm, even after sentinel lymph node biopsy. Why not teach patients how to do lymphodema massage? | Management of nerve damage/pain |
| **C6.4** | Are there ways to prevent/reduce scar stretching during healing after mastectomy? i.e. a less than 1mm incision width directly after surgery might end up as 5-10mm width scar once healed. | How can we minimise scarring after breast cancer surgery? |
| **C84.5** | What type of (physio)therapy is most useful to retain or regain full shoulder movement after breast surgery with removal of lymph nodes and what is the best timing for this? | What is the best way to offer physiotherapy to patients having breast cancer surgery? When should it be offered and what types of physiotherapy are the most helpful? |
| **C78.9** | * importance of expert physio |  |
| **C99.3** | Does face to face physiotherapy after surgery (single or multiple appointments) improve mobility outcomes compared to videos/leaflets about exercises? |  |
| **C113.6** | Physios right away for shoulder mobility |  |
| **C137.4** | And sending someone in to tell you what stretches to when your sitting in hospital gown and surgical gown. How are you expected to take it in. Maybe it’s to distract you but felt more like a tick ✔️List |  |
| **C170.15** | How soon after surgery can you begin to exercise/commence physiotherapy? |  |
| **C74.9** | Knowing how to manage cording and the role of physiotherapy would be useful. |  |
| **C73.5** | I was keen to know how quickly I could start doing things after surgery and what physio therapy exercises I could do and how soon. I wanted to know how soon I could shower and what precautions I needed to take. |  |
| **THEME 7: LONG TERM FOLLOW UP AND SUPPORT** | | |
| **C39.4** | What are the long-term effects of radiotherapy in terms of scar healing and pain levels? | What are the long-term effects of radiotherapy on the breast after surgery? |
| **C48.8** | Why are long term effects explained more clearly? E.g what to expect even 5 years down the line? | And what information and support do patients want about the possible long-term effects of breast cancer treatments and how can we best provide this? |
| **C127.5** | Would it be possible to provide some information on long term outcomes following breast surgery as other than the risk of cancer recurrence these do no seem to be discussed with patients? e.g. impact on arm movement, strength, random pain, longer term shape changes of the breast and also psychological impact? |  |
| **C143.3** | Understanding what is normal now, what are the concerns related post surgery and what should we be checking for. The implications of surgery should have been discussed more, long term numbness, pain. |  |
| **C125.4** | More information on side-effects would be good. I had no idea about the nerve damage and loss of sensation because of lymph node clearance until it happened. In the shorter term, information on spotting signs of infection while draining would have been helpful, and some indication of how much pain is normal and how much an indication that something is wrong. |  |
| **C74.11** | More information about risk of local recurrence and what to be aware of would be useful, particularly in relation to scar tissue post surgery. |  |
| **C166.3** | Post-op effects of pain/numbness at site circa 12-36 months after a lumpectomy |  |
| **C56.9** | Phantom breast pain following mastectomy, why is it not discussed prior to surgery? How common is phantom breast pain? How can it be managed? |  |
| **C86.5** | With the diep flap can this cause internal scarring and bowel problems later in life? |  |
| **C34.3** | No information on long term effects scar tightening and the numbness and pain that never goes away. |  |
| **C161.9** | How do you make sure quality of life is preserved post op - dealing with altered sensations and psychological issues and pain? |  |
| **A111.2** | Endocrine therapy effective but are not followed up or monitored for the common problems associated with these drugs. An endocrine follow up should be essential as cholesterol often becomes a problem, heart problems more likely ect. I sit on a HIV MDT and these patients are monitored twice a year for toxicity and general well-being . |  |
| **C15.4** | How can we be more honest with patients that treatment doesn’t stop after chemo, radio surgery? The hormone therapy has been extremely hard for me. I have had to try a number of drugs but they all have things in common and that is hot flushes and stiffness. I was pre menopausal before chemo and chemo put me in to an early menopause. Letrozole rendered me immobile. I also developed carpal tunnel syndrome which needed surgery. |  |
| **C170.11** | Are there any red flag symptoms to be aware of? |  |
| **C53.9** | How can patients access clear pathway of help for complications and side effects? (Had to pay privately for physio as GP wouldn’t help and neither would the breast care unit). | How can we ensure that patients with long-term problems after breast cancer surgery can easily access help and support? |
| **C145.2** | Follow up support after treatment would have benefitted me. At the end of my treatment, I was scared, worried and very anxious. This was because all the staff involved in my treatment had been wonderful; I felt as if I'd been wrapped up in cotton wool and taken care of in the most sensitive and caring way. However, after treatment, there was nothing ; it was a lonely, vulnerable and extremely emotional period for me. This has eased overtime, although the anxiety hasn't, but it was a very difficult transition from in-treatment to out-of-treatment and recovery. Also, in terms of advances of prognosis/survival post treatment, could there be a way of keeping women who've been through treatment informed? I've been given my survival chances in the first ten years following treatment but I don't know if that could change depending on research and just generally more been known about breast cancer treatments and prognosis. I don't know how to go about finding out. Seven years on, I still worry about it a lot. I see things on social media that lead to forum discussions, but it's all aimed at women whose cancers have advanced. I understand only too well that they need a lot of discussion and support and I hope that will never be me, but I don't know that and a support network of cancer survivors, like me, who are just getting on with their lives would be wonderful. | What psychological and practical support do patients who have been treated for breast cancer want in the years following their surgery? |
| **C49.12** | Do you think that your local health board provides adequate emotional support to patients undergoing breast cancer surgery in the long term? |  |
| **C29.6** | Supporting people who have issues well after treatment |  |
| **A33.1** | Support for including physical activity as a follow up guide and social prescribing to certain groups |  |
| **C161.7** | How should long term follow up be managed? | Why is there variation in how patients are followed up after breast cancer surgery and what way is best? |
| **C112.10** | Best form of follow up - community or back to hospital |  |
| **C53.6** | Why do some health trusts provide regular follow ups to check surgery sites and others do not? ( I have not been checked at all part from when the dressing was taken off 2 years ago. |  |
| **C11.3** | I was text book post op, all went to plan, longterm effects are numbness, feeling anxious about looking for lumps when you have had both breasts removed, i really need/look forward to being examined by my surgeon as I did not find my lump, even though i self examined thoroughly regularly, So the relief is enormous when this is done and i feel relaxed and less stressed |  |
| **C49.10** | How long should a breast cancer patient who has undergone a single or double mastectomy be followed up post surgery? |  |
| **C110.16** | Does long term follow up (for cancer survivors) improve outcomes for patients? |  |
| **C110.17** | In those who are diagnosed young there is no support after discharge at 10 year mark. I feel as though I have been left high & dry! Where/what is the support? |  |
| **A111.3** | After five years you are discharged... why? Cancer rarely goes away fully and for lobular patients the stealth with which it moves around should indicate that it is already distributed to other places via lymph glands at a cellular level. Finally, if no further follow up how do begin to understand this unique subtype? How can we tell when our cancer returns? There is no solid tumour to feel. Countless times I encounter medical professionals who have no idea about lobular and don’t want to listen as I am only the patient. This is what makes me an advocate. I read peer reviewed papers, attend conferences, can critique evidence. Have even put together a lobular charity to help other women understand this diagnosis. Education about lobular needs to be more widely considered. |  |
| **THEME 8: HIGH RISK PATIENTS AND SPECIAL GROUPS** | | |
| **C40.4** | Once preventative surgery is completed some sort of follow up with counselling should be completed. I have 3 close family members and they have been left to suffer the consequences of mishandled preconselling and 2 from bad bed side manner from their surgeon. Also giving them a point of contact post op would be nice instead of being dropped like a hot potato. | What psychological or emotional support do women want after risk-reducing surgery? |
| **A168.3** | Options for people at high risk, for managing that risk? | How can we best support patients who carry a breast cancer gene to make a decision about whether or not they would like risk reducing surgery, including discussing other options? |
| **A56.1** | Questions about the mental and emotional impact on patients who are diagnosed or at risk. In particular those of us without partners who don’t have that level of close emotional support. |  |
| **A171.1** | As someone with a confirmed gene mutation, what are the ways I can reduce risk? Is surgery the only option? |  |
| **B40.2** | If the woman isn't scanned she also does not see anyone to discuss what this means and what the future options are. For example, questions they might have on future procedures and timeliness for these. This could be fixed with a simple phone call ultimately. Ultimately I have the gene but as to my options for surgery, no one has discussed this with me so I can't recommend any research avenues. |  |
| **B40.3** | Also if a woman has breastfed for 6 months plus is there any lowering of her risk ie prolong the time before she should consider surgery. Family planning services should also be brought in to ensure that woman are counselled on the fact they want u to have finished ur family before surgery. |  |
| **A30.3** | Is mastectomy for younger women the best/only way to treat patients with a history of breast cancer in their family? |  |
| **A133.1** | Support is important As a man who has had breast cancer , the support for me was non existent . I was only offered support with women which was not what was wanted . More separate focusing on MEN | How can the experiences of men with breast cancer be improved? How can we ensure that they are offered all appropriate treatment options and tailored psychological support? |
| **A129.3** | Q. When the results are divulged two weeks after the initial appointment and biopsy, why is it that Doctors tell men, mastectomy is required, never breast conserving surgery always total mastectomy. |  |
| **B129.8** | Q. Why is reconstructive surgery never discussed with Male breast cancer patients. Why are so called “Dog Ears”, flaps, just left and no mention of removal is offered. |  |
| **C129.16** | Q. Why are men less likely to be consoled, or offered emotional support than ladies. |  |
| **C129.17** | Q. Why post treatment are men offered no follow up support, even in the way of information re external support groups. |  |
| **A129.1** | Q. What provision could be made to limit the additional stress men attending breast clinics might be feeling, surrounded by a totally pink environment, both visually and in written information, notice boards, waiting rooms, etc. |  |
| **A129.7** | Q. What can be done to totally include men in the prevention, diagnosis, treatment and aftercare of this disease, why is it female dominated, and men are rarely included, if they are it is tokenistic. |  |
| **B129.10** | Q. Why are men made to feel they can cope, and left with very little follow up or during treatment support. |  |
| **C129.15** | Q. Is it an assumption that breast cancer in men should automatically be treated as per the way females are treated . Anatomically we are totally different. |  |
| **A131.1** | Counselling is a big question particularly for men |  |
| **B129.9** | Q Why do health professionals think Male patients feel less body image issues than females. |  |
| **A170.4** | How does race/ethnicity impact risk factors/ outcomes? | How does ethnicity affect the choice and results of breast cancer surgery? |
| **C170.14** | Does race/ethnicity impact outcomes/risk factors/side effects. |  |

**Online resource 2: Number of participants including each question in their ‘top 10’ and overall question rank by Delphi Round**

|  | **Round 1**  **(n=237)** | | **Round 2**  **(n=170)** | |
| --- | --- | --- | --- | --- |
|  | **Top 10**  **N (%)** | **Round 1 Rank** | **Top 10**  **N (%)** | **Round 2 Rank** |
| *What psychological and practical support do patients who have been treated for breast cancer want in the years following their surgery? | 75 (31.7) | 2 | 97 (57.1) | 1 |
| How many women get lymphoedema after breast cancer surgery? Can it be prevented? What is the best way to detect and treat lymphoedema if it develops? | 76 (32.1) | 1 | 76 (44.7) | 2 |
| *What psychological or emotional support do patients having breast cancer surgery need? When is it needed and how can we improve the way that it is provided so that more patients can benefit? | 62 (26.2) | 5 | 72 (42.4) | 3 |
| Are there situations when the removal of the healthy opposite breast should be considered for psychological reasons in patients with breast cancer who have had a mastectomy but do not carry a breast cancer gene? | 64 (27.0) | 3 | 60 (35.3) | 4 |
| In patients having mastectomy for breast cancer who do not carry a breast cancer gene, should a balancing mastectomy for symmetry be offered at the same time or at a later date? What factors may influence this decision? | 64 (27.0) | 4 | 59 (34.7) | 5 |
| How can we best prepare patients for breast cancer surgery including aftercare (e.g. bras and dressings) and what to expect after the operation? What information do patients want and how should this be provided? | 47 (19.8) | 10 | 57 (33.5) | 6 |
| What is the best way to perform a mastectomy operation to achieve an aesthetic flat closure? How can we ensure that all surgeons are trained to perform this and can offer it routinely to patients who do not want breast reconstruction? | 58 (24.5) | 6 | 53 (31.2) | 7 |
| What information do patients want around the time of diagnosis to help them make decisions about their breast cancer treatment options and how should it be provided? | 52 (21.9) | 7 | 50 (29.4) | 8 |
| How can we ensure that all patients with breast cancer are offered and given fair and balanced information about all appropriate types of breast cancer surgery? How can we best support patients to make decisions about what option is best for them? | 48 (20.3) | 9 | 49 (28.8) | 9 |
| *What practical and emotional support do patients with breast cancer want at diagnosis and during treatment? | 46 (19.4) | 11 | 47 (27.7) | 10 |
| How can we best support patients who chose to stay flat after mastectomy for breast cancer? | 52 (21.9) | 8 | 45 (26.5) | 11 |
| How can we improve communication between patients with breast cancer and the clinical team? | 45 (19.0) | 14 | 45 (26.5) | 12 |
| How can we support healthcare professionals to routinely offer balancing mastectomy for symmetry as an alternative to breast reconstruction in patients who do not carry a breast cancer gene who have had a single mastectomy for breast cancer? | 45 (19.0) | 15 | 43 (25.3) | 13 |
| How can we support patients to be fully involved in decisions about their breast cancer treatment? How can this support be tailored according to the patient’s wish to be involved? | 45 (19.0) | 13 | 37 (21.8) | 14 |
| What are the long-term effects of radiotherapy on scarring and pain? | 46 (19.4) | 12 | 35 (20.6) | 15 |
| How can we provide better information about less common forms of breast cancer (e.g. lobular cancers) including how this may affect breast cancer surgery and other treatments? | 41 (17.3) | 19 | 34 (20.0) | 16 |
| What information and support do patients want about the possible long-term effects of breast cancer surgery? What is the best way to provide this so that patients who experience problems can most easily access help and support? | 39 (16.5) | 25 | 32 (18.8) | 17 |
| What are the short and long-term outcomes of mastectomy surgery and how does mastectomy affect patients’ quality of life? | 43 (18.1) | 17 | 31 (18.2) | 18 |
| Why do seromas (collections of fluid that build up under the skin), develop after breast cancer surgery? Can they be avoided and when do they need to be drained? | 43 (18.1) | 18 | 30 (17.7) | 19 |
| Why is there variation in the types of breast cancer treatment (including surgery) offered in different areas of the UK and how can this be addressed? | 41 (17.3) | 20 | 29 (17.1) | 20 |
| What is the best way to offer physiotherapy to patients having breast cancer surgery? When should it be offered and what types of physiotherapy are the most helpful? | 40 (16.9) | 23 | 26 (15.3) | 21 |
| How can we improve the support offered by specialist nurses to patients with breast cancer? | 35 (14.8) | 27 | 26 (15.3) | 22 |
| What information and support to patients need to recognise that they have developed a problem (complication) after their breast cancer surgery and know who to contact so they can be effectively assessed and treated? | 39 (16.5) | 24 | 25 (14.7) | 23 |
| What are the short & long-term cancer-related outcomes after breast conserving surgery+radiotherapy vs mastectomy? Which operation has the lowest risk of the cancer returning? | 44 (18.6) | 16 | 24 (14.1) | 24 |
| What is the best way to treat patients who develop nerve pain after breast cancer surgery? | 40 (16.9) | 22 | 24 (14.1) | 25 |
| How can we best tailor information about breast cancer treatments for individual patients so that it is personalised for them and their circumstances? | 35 (14.8) | 26 | 23 (13.5) | 26 |
| How can we make prostheses better and easier to use after mastectomy for breast cancer? | 34 (14.4) | 28 | 22 (12.9) | 27 |
| What is the best way to manage pain after breast cancer surgery? How can we avoid patients developing long-term (chronic) pain? | 30 (12.7) | 30 | 18 (10.6) | 28 |
| What is the best way to provide support for patients who have had breast surgery after they have been discharged from hospital? | 29 (12.2) | 31 | 17 (10.0) | 29 |
| How can we reduce waiting lists and improve access to delayed breast reconstruction for patients who had mastectomy for breast cancer? | 27 (11.4) | 35 | 17 (10.0) | 30 |
| Why is there variation in whether women who do not carry a breast cancer gene are offered a balancing mastectomy as an alternative to breast reconstruction after a single mastectomy for breast cancer and how can this be addressed? | 27 (11.4) | 36 | 17 (10.0) | 31 |
| Why is there variation in whether patients treated for breast cancer are followed up by their surgeons in the UK? What type of follow up is best and how should this be provided? | 33 (13.9) | 29 | 16 (9.4) | 32 |
| Would images including 3D scanning or computer simulations showing the likely cosmetic outcomes of different operations help patients make better decisions and improve their satisfaction with breast cancer surgery? | 40 (16.9) | 21 | 14 (8.2) | 33 |
| How can we minimise scarring after breast cancer surgery? | 28 (11.8) | 34 | 14 (8.2) | 34 |
|  | **Round 1**  **(n=237)** | | **Round 2**  **(n=170)** | |
|  | **Top 10**  **N (%)** | **Round 1 Rank** | **Top 10**  **N (%)** | **Round 2 Rank** |
| Why is there variation in whether (or not) women are offered breast reconstruction surgery? How can we ensure that all patients have equal access to appropriate breast reconstruction options, either at the time of surgery or at a later date? | 25 (10.6) | 37 | 12 (7.1) | 35 |
| What is the role of charities and support groups for patients with breast cancer and when are they most useful? | 18 (7.6) | 46 | 12 (7.1) | 36 |
| Are women who have opted for balancing mastectomy for symmetry satisfied with their decision and how do they feel about the results of their surgery? How does this compare to women who opted for breast reconstruction? | 18 (7.6) | 50 | 12 (7.1) | 37 |
| What is the best way to support the partners/families of patients diagnosed with breast cancer? | 28 (11.8) | 32 | 11 (6.5) | 38 |
| Does breast reconstruction surgery affect future breast cancer treatments or long-term cancer outcomes? | 28 (11.8) | 33 | 11 (6.5) | 39 |
| How can we improve continuity of care between hospital and the community for patients having breast cancer surgery? | 20 (8.4) | 44 | 11 (6.5) | 40 |
| How should patients having breast cancer surgery be cared in hospital after their operation? Do patients need to stay in hospital overnight after breast cancer surgery? | 25 (10.6) | 39 | 10 (5.9) | 41 |
| What are the short and long-term outcomes of different types of breast reconstruction operation and how do they affect patient’s quality of life and well-being? | 24 (10.1) | 40 | 10 (5.9) | 42 |
| Why is there variation in whether a surgical drain is used after breast cancer surgery? How are they best looked after and are they always needed? | 25 (10.6) | 38 | 9 (5.3) | 43 |
| How can we best support patients with breast cancer who do not have a social support network? | 20 (8.4) | 43 | 9 (5.3) | 44 |
| How can the experiences of men with breast cancer be improved? How can we ensure that they are offered all appropriate treatment options and tailored psychological support? | 11 (4.6) | 58 | 9 (5.3) | 45 |
| How can we ensure all patients are given fair and balanced information about all types of breast reconstruction including why some may not be suitable? What information do patients want and how should this be provided? | 19 (8.0) | 45 | 8 (4.7) | 46 |
| How can we ensure that all appropriate patients are offered and given fair and balanced information about oncoplastic breast conserving surgery (operations that combine lumpectomy to remove the cancer with plastic surgical techniques to reshape the breast) including symmetrisation (balancing surgery) to the opposite breast if needed? | 14 (5.9) | 55 | 8 (4.7) | 47 |
| What information and support do patients with breast cancer want to help them make decisions about whether to have chemotherapy before or after surgery? | 12 (5.1) | 57 | 8 (4.7) | 48 |
| What psychological or emotional support do women want after risk-reducing How can we best support individuals at high risk of developing breast cancer to make decisions about risk-reducing surgery, including discussing other options? | 10 (4.2) | 60 | 8 (4.7) | 49 |
| How does radiotherapy affect the outcomes of breast reconstruction surgery? | 21 (8.9) | 42 | 7 (4.1) | 50 |
| How can we reduce the risk of patients developing problems after implant-based breast reconstruction in the short and long term? | 18 (7.6) | 49 | 7 (4.1) | 51 |
| What is the value of a ‘buddy system’ for patients newly diagnosed with breast cancer? | 21 (8.9) | 41 | 5 (2.9) | 52 |
| How do the outcomes of breast reconstruction performed at the time of mastectomy compare with those of breast reconstruction performed at a later date? How do both options affect the quality of life and well-being of patients with breast cancer? | 18 (7.6) | 48 | 4 (2.4) | 53 |
| What is the best way to support patients with learning disabilities to make decisions about their breast cancer treatment options? | 16 (6.8) | 51 | 4 (2.4) | 54 |
| Are there any alternatives to implant-based reconstruction for women who do not have enough tissue for a tissue-based reconstruction? | 13 (5.5) | 56 | 4 (2.4) | 55 |
| How does ethnicity affect the choice and results of breast cancer surgery? | 7 (3.0) | 62 | 4 (2.4) | 56 |
| What is the best order of breast cancer treatments (surgery, chemotherapy and/or radiotherapy) for women wishing to have immediate breast reconstruction? | 18 (7.6) | 47 | 3 (1.8) | 57 |
| Why is there variation in the types of breast reconstruction operation that patients after offered after mastectomy in the UK and how can this be addressed? | 15 (6.3) | 52 | 3 (1.8) | 58 |
| Is nipple sparing mastectomy a safe option for patients with breast cancer having breast reconstruction? | 10 (4.2) | 59 | 3 (1.8) | 59 |
| How can we best provide support for patients having chemotherapy before surgery during their treatment? | 14 (5.9) | 53 | 2 (1.2) | 60 |
| Should radiotherapy be given before surgery in patients with breast cancer? | 14 (5.9) | 54 | 2 (1.2) | 61 |
| Why do different surgeons use different dressings after breast cancer surgery? Which are best and when should they be removed? | 8 (3.4) | 61 | 1 (0.6) | 62 |
| Is it possible to avoid the need for further surgery in patients having breast conservation procedures where not all the cancer is completely removed? | 4 (1.7) | 63 | 1 (0.6) | 63 |

*merged into single question for workshop: ‘What psychological, practical and emotional support do patients with breast cancer want, and when is it needed, and how can we improve the way that it is provided so that more patients can benefit?’

**Online resource 3: 25 questions discussed at the final prioritization workshop**

A Are there situations when the removal of the healthy opposite breast should be considered for psychological reasons in patients with breast cancer who have had a mastectomy but do not carry a breast cancer gene?

B How can we best prepare patients for breast cancer surgery including aftercare (e.g. bras and dressings) and what to expect after the operation? What information do patients want and how should this be provided?

C How can we best support patients who chose to stay flat after mastectomy for breast cancer?

D How can we best tailor information about breast cancer treatments for individual patients so that it is personalised for them and their circumstances?

E How can we ensure that all patients with breast cancer are offered and given fair and balanced information about all appropriate types of breast cancer surgery? How can we best support patients to make decisions about what option is best for them?

F How can we improve communication between patients with breast cancer and the clinical team?

G How can we improve the support offered by specialist nurses to patients with breast cancer?

H How can we make prostheses better and easier to use after mastectomy for breast cancer?

I How can we provide better information about less common forms of breast cancer (e.g. lobular cancers) including how this may affect breast cancer surgery and other treatments?

J How can we support healthcare professionals to routinely offer balancing mastectomy for symmetry as an alternative to breast reconstruction in patients who do not carry a breast cancer gene who have had a single mastectomy for breast cancer?

K How can we support patients to be fully involved in decisions about their breast cancer treatment? How can this support be tailored according to the patient’s wish to be involved?

L How many women get lymphoedema after breast cancer surgery? Can it be prevented? What is the best way to detect and treat lymphoedema if it develops?

M In patients having mastectomy for breast cancer who do not carry a breast cancer gene, should a balancing mastectomy for symmetry be offered at the same time or at a later date? What factors may influence this decision?

N What are the long-term effects of radiotherapy on scarring and pain?

O What are the short and long-term cancer-related outcomes after breast conserving surgery and radiotherapy vs mastectomy? Which operation has the lowest risk of the cancer returning?

P What are the short and long-term outcomes of mastectomy surgery and how does mastectomy affect patients’ quality of life?

Q What information and support do patients want about the possible long-term effects of breast cancer surgery? What is the best way to provide this so that patients who experience problems can most easily access help and support?

R What information and support to patients need to recognise that they have developed a problem (complication) after their breast cancer surgery and know who to contact so they can be effectively assessed and treated?

S What information do patients want around the time of diagnosis to help them make decisions about their breast cancer treatment options and how should it be provided?

T What is the best way to offer physiotherapy to patients having breast cancer surgery? When should it be offered and what types of physiotherapy are the most helpful?

U What is the best way to perform a mastectomy operation to achieve an aesthetic flat closure? How can we ensure that all surgeons are trained to perform this and can offer it routinely to patients who do not want breast reconstruction?

V What is the best way to treat patients who develop nerve pain after breast cancer surgery?

W What psychological, practical, and emotional support do patients with breast cancer want, and when is it needed, and how can we improve the way that it is provided so that more patients can benefit?

X Why do seromas (collections of fluid that build up under the skin), develop after breast cancer surgery? Can they be avoided and when do they need to be drained?

Y Why is there variation in the types of breast cancer treatment (including surgery) offered in different areas of the UK and how can this be addressed?
